# Numerical calculation and analysis of filtration performance of an effective novel structural fiber for PM_2.5_

**DOI:** 10.1371/journal.pone.0240941

**Published:** 2020-10-22

**Authors:** Hui Yang, Hui Zhu, Haiming Fu

**Affiliations:** 1 College of Environmental Science and Engineering, Donghua University, Shanghai, China; 2 College of Energy and Building Environment, Guilin University of Aerospace Technology, Guilin, China; Shanghai Jiao Tong University Medical School Affiliated Ruijin Hospital, CHINA

## Abstract

In this study, a novel fiber with slit-crescent-shaped cross-section is proposed to enhance the filtration performance of PM_2.5_ in fibrous filtration. The collection efficiency of this fiber is simulated by using a Brownian dynamics simulation technique, and its filtration pressure drop is obtained by numerically solving Navier-Stokes equation with Fluent software. A parametric study is performed to improve the optimum filtration performance of the slit-crescent-shaped fiber via adjusting its structural parameters (dimensionless center-to-center spacing and slit width). Results indicate that at the optimal condition, i.e., when dimensionless slit width ranges from 0.2 to 0.4, collection efficiency is enhanced by 13.1%–101.1% relative to the circular fiber for particles ranging from 0.1μm to 2.5μm for the slit-crescent-shaped fiber under various dimensionless center-to-center spacing, and filtration pressure drop is reduced by up to 14.4%. In addition, quality factor is introduced to evaluate the comprehensive filtration performance of the slit-crescent-shaped fiber with different structural parameters, and results show that large dimensionless slit width and small dimensionless center-to-center spacing lead to a much higher quality factor than the circular fiber, especially for particles lager than 0.5μm. The numerical results obtained in this work are conducive to designing high efficiency fibrous filters.

## 1. Introduction

Respirable aerosol particles (PM_2.5_, particle diameter ≤2.5μm) emitted from nature and human activities are harmful and can damage the respiratory immune function as well as the central nervous and cardiovascular systems of human body [1, 2]. Fibrous filtration is the most widely used air filtration technique for removal of PM_2.5_ from gas stream, and is applied in a variety of residential, medical, industrial air conditioning systems and personal respirators (e.g., N95/N99) [3]. The comprehensive filtration performance of fibrous filter is typically evaluated by quality factor (*QF*) [4], which is defined as *QF* = −ln(1−*E*)/Δ*p*, where *E* is collection efficiency and Δ*p* is pressure drop. A higher *QF* fibrous filter should perform with lower pressure drop and much higher collection efficiency. In order to improve quality factor of fibrous filters, substantial efforts have been conducted by many researchers, and many achievements have been made. Such researches include composites of nanometer and micrometer fibers [5–8], optimization of fiber arrangement [9, 10], fibrous media morphology modification [11–13], and so on.

With the recent advancements in fiber manufacturing technology, noncircular cross-sectional fibers, for instance, rectangular, elliptical, triangular, trilobal, etc., have emerged as a promising fiber media with potentially higher collection efficiency and quality factor than conventional circular fibers [14–16]. Many researches have been studying collection efficiency and pressure drop of rectangular fibers [17–19]. Such studies revealed that rectangular fibers had a higher diffusional collection efficiency than circular ones, however, their pressure drops are also higher. Numerical simulations by Hosseini and Vahedi Tafreshi [20] indicated that the difference between collection efficiency of square fibers and circular ones were negligibly small for particles ranging from 0.1μm to 0.7μm, however, filtration pressure drop of the former is higher than the later.

Flow field, pressure drop and particle collection efficiency of elliptical fibers have also been investigated [21–26], and results show that these fibers are much better than conventional circular fibers in removing nanoparticles dominated by Brownian diffusion, however, their pressure drops are also higher in the case of large orientation angle and aspect ratio. Recently, Jin et al. [27] numerically investigated filtration characteristics of randomly arranged elliptical fibers as well as circular fibers, and found that elliptical fibers had a lower quality factor than that of circular ones for sub-microparticles (0.1μm–1μm).

Experimental work by Sánchez et al. [14] proved that trilobal polyimide filters had a higher collection efficiency than circular polyester filters for particles ranging from 0.7μm to 1.2μm when filtration velocity is 0.1m/s, while shows a lower collection efficiency at the high filtration velocity of 0.2m/s. Gu et al. [16] investigated the filtration performance of fibers with Y-shaped and circular section by numerical and experimental methods, and concluded that retention of PM_2.5_ with Y-shaped fibers is better than that with circular fibers. Tafreshi et al. [20, 28] numerically simulated the filtration process of trilobal fibers, and found that despite having a higher collection efficiency than circular fibers for sub-microparticles (0.1μm–1μm), trilobal fibers had a lower quality factor because of its much higher pressure drop. Similar researches have also been reported in other studies [15, 29].

Although noncircular fibers mentioned above usually have a higher collection efficiency than conventional circular fibers for PM_2.5_, pressure drop of noncircular fibers is also generally higher, which lead to larger energy consumption and poorer quality factor than circular fibers. Thus, high-efficiency and low-energy consumption fibrous filters are still highly desirable.

In this study, a novel fiber with slit-crescent-shaped cross-section is proposed to lower filtration pressure drop and enhance collection efficiency of PM_2.5_. A numerical method is employed to investigate particle filtration process of slit-crescent-shaped fibers. Particles smaller than 0.1μm where Brownian diffusion is predominant can be efficiently removed by conventional circular fibers, while those ranging from 0.1μm to 2.5μm with relatively weak effects of Brownian diffusion and inertia impaction have a relatively low collection efficiency when collected by circular fibers. Therefore, a representative particle size range, i.e., 0.1μmδ *d*_p_ δ2.5μm, is selected to study the effects of structural parameters (dimensionless center-to-center spacing and dimensionless slit width) on filtration performance of slit-crescent-shaped fibers. The current work aims to present a new kind of non-circular cross-sectional fiber which can achieve a much higher quality factor for PM_2.5_ filtration, hoping to contribute to fibrous filter design and improvement of filtration performance in practical engineering.

## 2. Computational model

### 2.1 Fluid flow model

**Fig 1:**
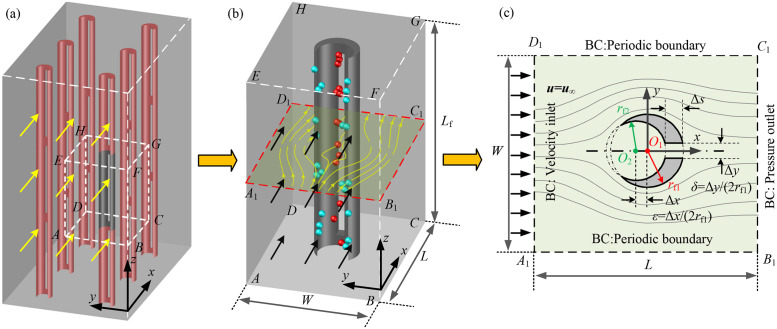
Simulation model and computational domain. (a) Model of parallel fiber arrangement, (b) Simulation model, (c) Schematic of the slit-crescent-shaped fiber.

We consider an arrangement made up of equally spaced slit-crescent-shaped fibers paralleling to each other with the axis of these fibers perpendicular to the flow, and thus the flow field around each fiber is completely consistent, as shown in Fig 1(a). As the slit-crescent-shaped fiber is infinitely long along the Z-axis, a representative cuboid domain *ABCDEFGH* in Fig 1(a) is adopted for flow field calculation, as shown in Fig 1(b). In order to clearly identify the structural parameters of the slit-crescent-shaped fiber, a 2D cutting plane *A*_1_*B*_1_*C*_1_*D*_1_ is taken out from Fig 1(b), as shown in Fig 1(c).

As illustrated in Fig 1, *L*_f_, *W* and *L* are height, width and length of the computational domain respectively (*W = L*), *r*_f1_ and *r*_f2_ are circumscribed circle radius and inscribed circle radius of the slit-crescent-shaped fiber correspondingly, the circumscribed circle radius remains constant (*r*_f1_ = 5μm), Δ*x* and Δ*y* are the center-to-center spacing of the circumscribed circle and inscribed circle as well as the slit width, *ε* is the dimensionless center-to-center spacing expressed as *ε* = Δ*x* /(2*r*_f1_) and *δ* is the dimensionless slit width defined as *δ* = Δ*y* /(2*r*_f1_), Δ*s* is the slit depth, which depends on *ε*, and Δ*s* increases with the increasing *ε*.

Generally, the fluid flowing through the fibrous filter is laminar as the fiber Reynolds number is much smaller than unity (Re<<1), as a consequence the flow across the fiber viewed in this paper can be simplified to a three-dimensional incompressible steady viscous laminar flow. The vectorial form of the continuity and momentum equations are respectively given as [30]:

$$\nabla \cdot \vec{u}=0$$ (1)

$$(\vec{u}\cdot \nabla )\vec{u}=-\frac{1}{\rho }\nabla p+\nu \cdot \nabla^{2}\vec{u}$$ (2)

where *ū* is fluid velocity vector, *ν* is fluid kinematic viscosity, *ρ* is fluid density, *p* is fluid pressure.

The numerical solutions of the continuity and momentum equations are carried out with finite volume method implemented in Fluent software. To ensure accuracy of numerical calculations, double precision solver is employed. Second order upwind scheme and SIMPLEC algorithm are adopted for control equations discretization and pressure-velocity coupling, respectively. Convergence criterion is set as 10^−6^ for continuity and momentum equations. The boundary conditions are presented in Fig 1(c). Uniform velocity inlet boundary condition and pressure outlet boundary condition are applied to inlet and outlet of the computational domain separately, and periodic boundary condition is considered for the sides of the domain [9]. For the air flow across the microfiber surface in this paper, a no-slip boundary condition is specified at the fiber surface [31].

### 2.2 Particle motion equation

The steady-state particle filtration process is considered, that is, the influence of deposited particles on flow field is ignored. The dominant forces exerting on a particle include air drag force and Brownian force, therefore, external field forces, such as electrostatic force and gravity, are not taken into consideration. Additionally, for a dilute gas-particle low velocity flow, particle rebound effect and particle-particle interaction can also be ignored in this work [32].

Under the above assumptions, the vectorial form of the particle motion equation can be written as [33]:

$$m_{\text{p}}\frac{d\vec{\upsilon }}{dt}={\vec{F}}_{\text{d}}+{\vec{F}}_{\text{b}}$$ (3)

$${\vec{F}}_{\text{d}}=\frac{3\pi \mu d_{\text{p}}}{C_{\text{c}}}(\vec{u}-\vec{\upsilon })$$ (4a)

$${\vec{F}}_{\text{b}}=m_{\text{p}}\cdot \vec{\zeta }\sqrt{\frac{\pi S_{0}}{\Delta t}}$$ (4b)

where, *m*_p_ is particle mass, $\vec{\upsilon }$ is particle velocity vector, *t* is time, ${\vec{F}}_{\text{d}}$ and ${\vec{F}}_{\text{b}}$ are drag force and Brownian force, *μ* is air kinematic viscosity, *d*_p_ is particle diameter, *C*_*c*_ is Cunningham slip correction factor [34], $\vec{\zeta }$ is independent Gaussian random number with zero mean and unit-variance, Δ*t* is particle time interval and *S*_0_ is spectral intensity of noise [35] defined by

$$S_{0}=\frac{216\mu \cdot \kappa_{\text{B}}T}{\pi^{2}\rho_{\text{p}}^{2}d_{\text{p}}^{5}C_{\text{c}}}$$ (5)

where *κ*_B_ is Boltzmann constant, *κ*_B_×1.38×10^−23^J/K, *T* is air temperature, *ρ*_p_ is particle density.

The discrete phase model (DPM) is employed in Fluent to track the particle trajectories around the fiber. The Brownian force is programmed in the form of a UDF for Fluent program [36]. Moreover, Fluent treats a particle as a point particle (i.e., a mass point) when particle motion trajectory is solved, and the collision behavior is determined when the particle center is in contact with the wall surface. Hence, DPM in Fluent can only calculate particle collection due to inertial impaction and Brownian diffusion rather than interception. To include the interception effect in our simulation, a UDF is developed to modify DPM module implemented in Fluent. The UDF subroutine will continuously monitor every particle, if the distance between the particle’s center and the fiber surface is less than one particle radius, the particle is considered as being captured and the current particle trajectory calculation is then terminated.

## 3. Results and discussion

In this work, effects of dimensionless center-to-center spacing and dimensionless slit width on pressure drop and particle collection efficiency of slit-crescent-shaped fibers are investigated numerically. Main parameters and their corresponding values adopted in the simulation are listed in Table 1.

**Table 1:** Values of parameters used in simulations.

Parameter	Values
Fiber circumscribed circle diameter *d*_f1_ / μm	10
Fiber length *L*_f_ / μm	50
Dimensionless center-to-center spacing *ε*	0.05–0.3
Dimensionless slit width *δ*	0.05–0.4
Particle diameter *d*_p_ */* μm	0.1–2.5
Filtration velocity *u*_∞_ / m·s^−1^	0.05
Air viscosity *μ* / Pa·s	1.82×10^−5^
Air pressure *p* / Pa	1.013×10^5^
Air temperature *T* / K	300

### 3.1 Validation of present model

#### 3.1.1 Grid independence verification

**Fig 2:**
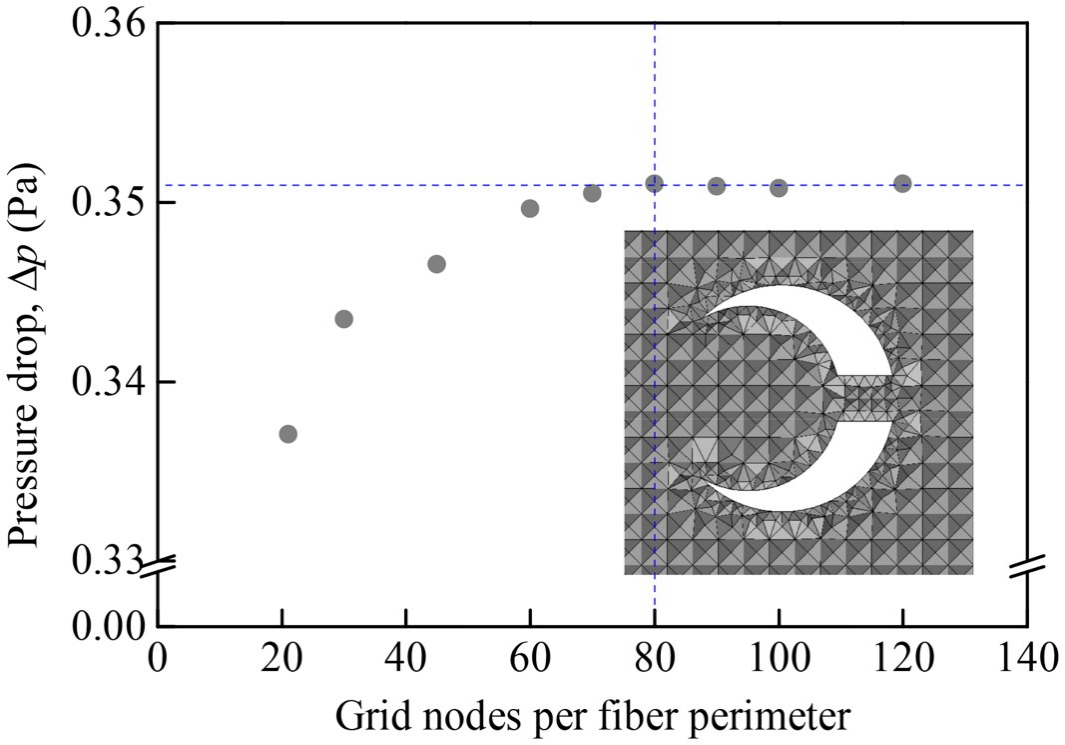
Effects of mesh density per fiber perimeter on pressure drop of the slit-crescent-shaped fiber.

The computational domain shown in Fig 1(b) is meshed using tetrahedral elements with fine grids within the vicinity of the fiber wall and coarser grids away from the fiber wall. To ensure that numerical calculation results are mesh-independent, we take a slit-crescent-shaped fiber with dimensionless center-to-center spacing *ε* = 0.15 and dimensionless slit width *δ* = 0.2, as an example, the fiber is meshed with different grid numbers on its perimeter. The pressure drop through the fiber with different grid numbers are calculated, and the results are presented in Fig 2.

It is evident that pressure drop gradually reaches a plateau as mesh density increases, the difference between 80 and 120 grids can be ignored. Thus, 80 grid nodes around the slit-crescent-shaped fiber is set for our numerical simulation.

#### 3.1.2 Validation of Brownian diffusion subroutine

According to the classical theory of Brownian movement, the mean displacement of particles due to Brownian diffusion is proportional to the square root of time *t* and the particle Brownian diffusion coefficient *D* [37], that is

$$\langle x_{i}(t)\rangle =\sqrt{2D\cdot t}$$ (6)

where 〈*x*_*i*_(*t*)〉 is the square root of the arithmetic mean of the squares of particle displacements in *i*-direction.

To validate the accuracy of the Brownian diffusion subroutine developed in this simulation, we consider a square simulation domain with quiescent air in Cartesian coordinate. N particles with given diameter are injected into the center of the simulation domain, denoted as *x*_*i*,*j*_ (0) = 0, *u*_*i*,*j*_ (0) = 0, *j* = 1, ⋯, *N*, and their displacement *x*_*i*,*j*_ (*t*) and time *t* per time step are recorded. Then, 〈*x*_*i*_(*t*)〉 is calculated by [37]

$$\langle x_{i}(t)\rangle ={\left[\frac{1}{N}\sum_{j=1}^{N}{\left(x_{i,j}(t)-x_{i,j}(0)\right)}^{2}\right]}^{1/2}$$ (7)

**Fig 3:**
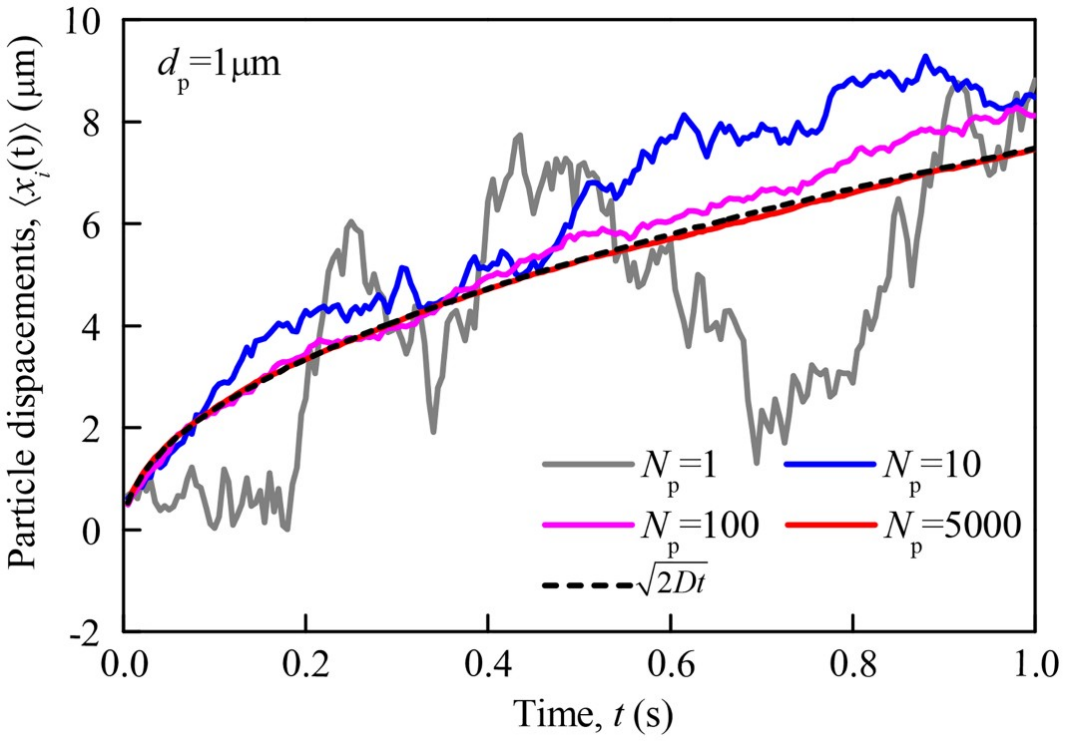
Verification of Brownian diffusion numerical model.

Fig 3 presents a case of such mean displacement calculation results in *x*-direction with particle diameter *d*_p_ = 1μm, time *t* = 1s, time step Δ*t* = 5ms and air temperature *T* = 300K. In Fig 3, the theoretical solution of mean displacement calculated from Eq (6) is also included. It can be seen that the Brownian movement of a single particle (ie, *N* = 1) exhibits significant random fluctuation effects, which is quite different from theoretical solutions. Furthermore, the difference between the mean motion displacement of the particles group calculated by Eq (7) and the theoretical solutions decreases with increasing statistical particle number. When the number of statistical particles increases to *N* = 5000, the difference between our results and the theoretical values can be negligible, which reveals that our Brownian diffusion subroutine has been correctly implemented.

#### 3.1.3 Validation of particle number density at the inlet

Collection efficiency of the slit-crescent-shaped fiber is determined by particle motion trajectory, which is affected by flow field and external forces exerting on the particle. We consider Brownian diffusion, interception and inertia impaction simultaneously in our calculation, so the dominant forces exerting on a particle include the air drag force and the Brownian force. For a fixed flow field and particle diameter, air drag force remains constant; while Brownian force depends Gaussian random number, and is a random variable. This means that motion trajectories of particles emitted from the same position are different from each other for a fixed flow field and particle diameter.

**Fig 4:**
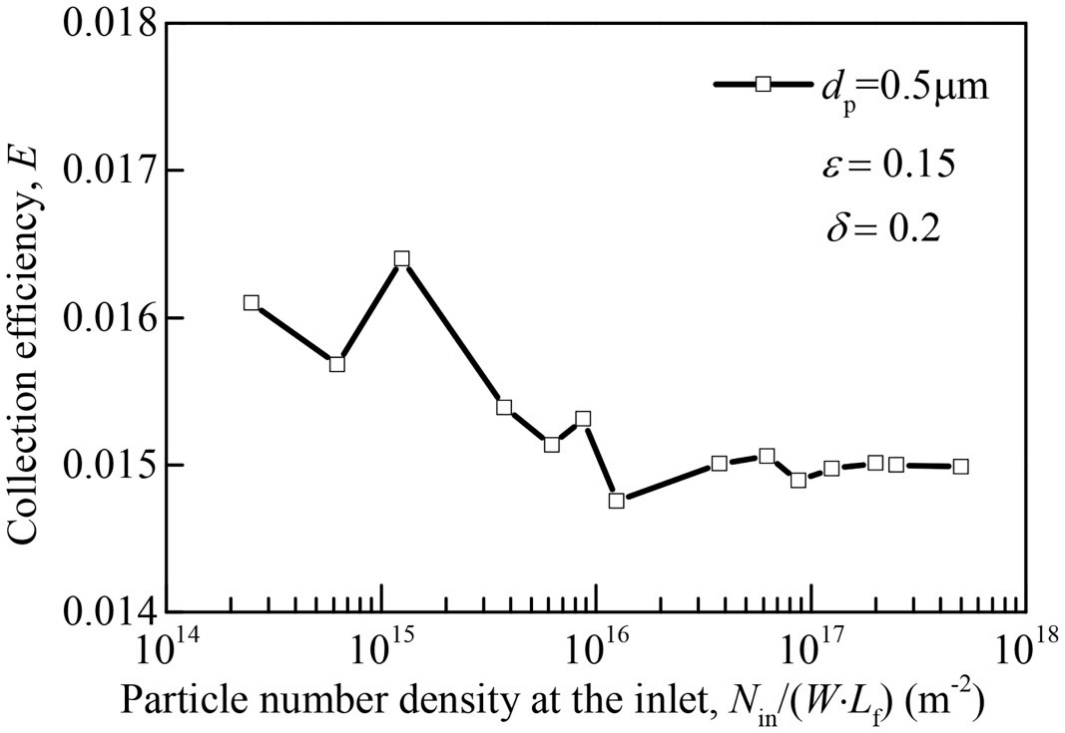
Influence of particle number density introduced at the inlet on collection efficiency of a typical slit-crescent-shaped fiber simulated in this study. *N*_in_ represents the number of injected particles from inlet of the domain, *ε* = 0.15, *δ* = 0.2 and *d*_p_ = 0.5 μm.

To reduce statistical fluctuations due to Brownian diffusion and achieve accurate collection efficiency and quality factor predictions, we release a large number of particles at the inlet of the domain and track their trajectory simultaneously. Influence of particle number density *N*_in_/(*W*∙*L*_f_) adopted at the inlet on collection efficiency *E* is presented in Fig 4. It can be seen that collection efficiency is independent of particle number density at the inlet when *N*_in_/(*W*∙*L*_f_) >1×10^17^ m^-2^, so this particle number density is adopted for collection efficiency calculation.

#### 3.1.4 Validation of particle collection efficiency

**Fig 5:**
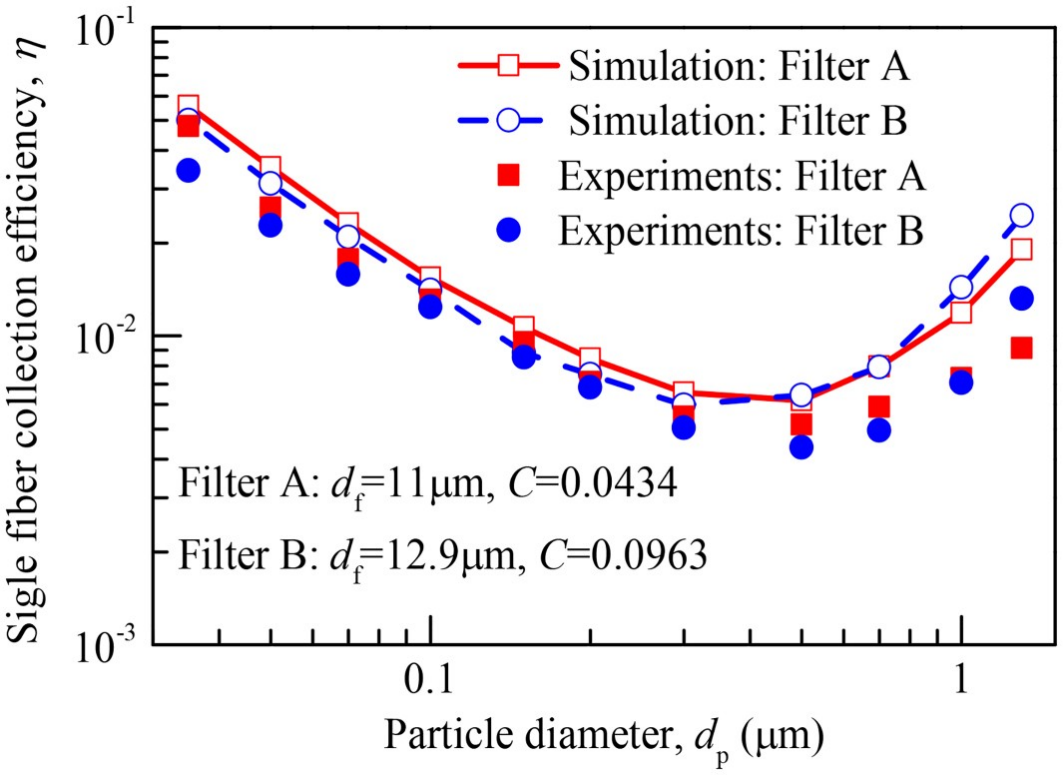
Comparisons of simulated collection efficiencies of circular fiber and experimental results. *d*_f_ represents fiber diameter, *C* represents fiber packing density.

Validation exercises are performed to ensure the reliability of our numerical model. The single fiber collection efficiency (*η*) of the circular fiber calculated by the same numerical method as the slit-crescent-shaped fiber is compared with the experimental data of Lee et al. [38], and the results are shown in Fig 5. As shown in Fig 5, the single fiber collection efficiencies predicted by our numerical model are in good agreement with the experimental results. Hence, this comparison validates our numerical model well.

### 3.2 Flow field and particle trajectories

**Fig 6:**
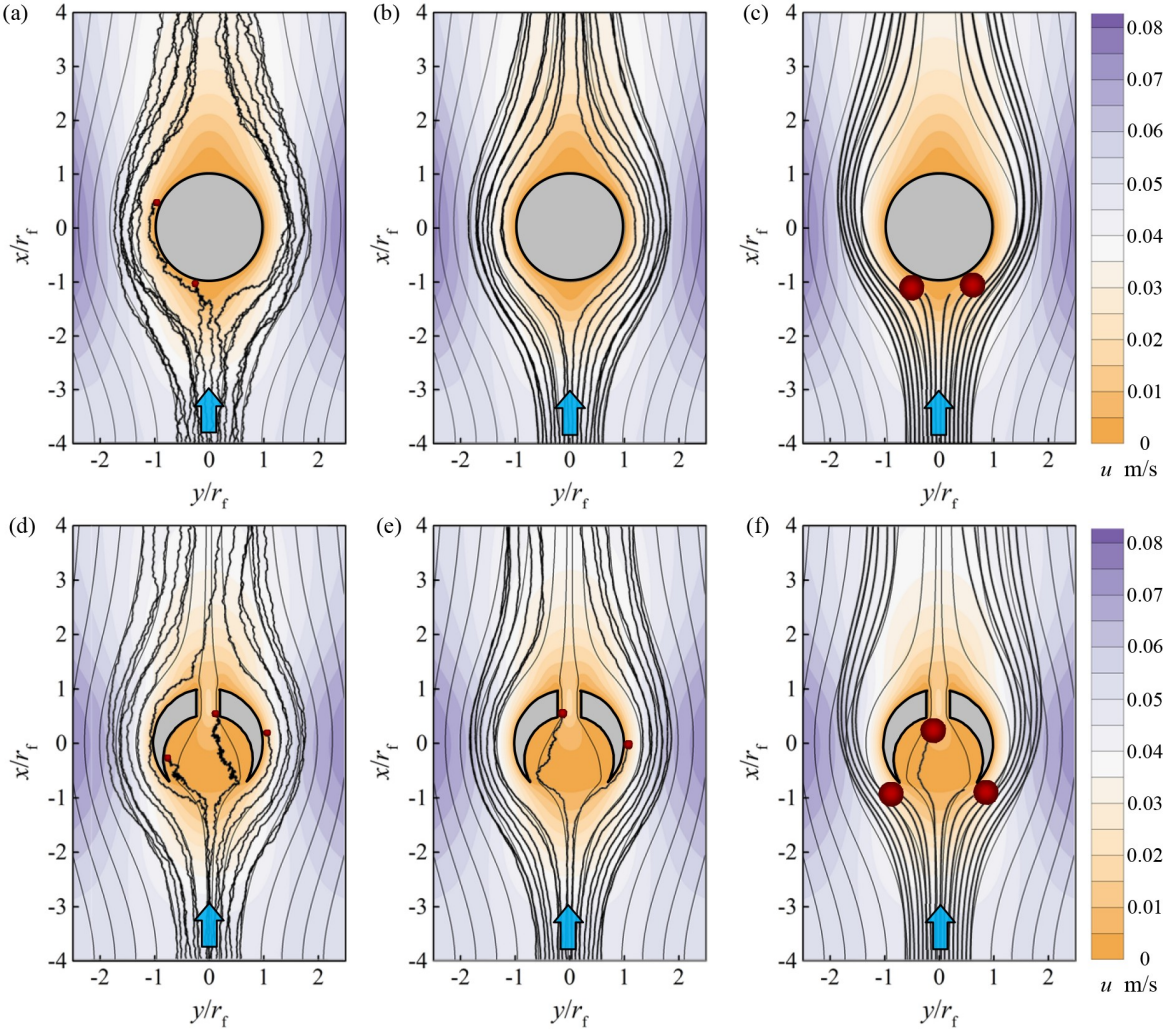
Flow field and particle trajectories. (a, b, c) Around the circular fiber, (d, e, f) Around the slit-crescent-shaped fiber with *ε* = 0.15, *δ* = 0.2, (a, d) *d*_p_ = 0.1μm, (b, e) *d*_p_ = 0.5μm, (c, f) *d*_p_ = 2.5μm.

Flow fields around the slit-crescent-shaped fiber with *ε* = 0.15 and *δ* = 0.2 are simulated and discussed in Fig 6, together with particle motion trajectories (*d*_p_ = 0.1μm, 0.5μm, 2.5μm). Flow fields and particle trajectories around the circular fiber are also shown in Fig 6 for comparison. The background contour represents velocity field, while the light gray smooth curve and the black curve denote streamlines and particle trajectories, respectively.

In Fig 6, the fluid flowing over the slit-crescent-shaped fiber is divided into two parts. The main part of the fluid passes around the fiber with a higher velocity, which is similar to the circular fiber, while the rest flows through the inner cavity with much smaller velocity than mean filtration velocity, which is significantly different from the circular fiber. Due to these low-speed particle-laden flows in the inner cavity of the slit-crescent-shaped fiber, Brownian diffusion of particles entering this region becomes stronger under such a small velocity. Such increased diffusion ability attenuates the particle carrying capacity of the fluid, and effects of random fluctuation of these particles are enhanced, leading to an increase in probability in the particle deposition, as shown in Fig 6(d)–6(f). This means that the novel slit-crescent-shaped fiber can efficiently improve the particle collection capacity.

By comparing the motion trajectories of particles with three different sizes (*d*_p_ = 0.1μm, 0.5μm, 2.5μm), it can be seen that the particle collection performance improvement by the slit-crescent-shaped fiber for intermediate particle (*d*_p_ = 0.5μm) is more notable than for fine particle (*d*_p_ = 0.1μm) and large particle (*d*_p_ = 2.5μm) when compared with the circular fiber. This leads to the conclusion that the slit-crescent-shaped fiber is more effective in removing intermediate particles.

### 3.3 Deposited particle distribution on the fiber surface

**Fig 7:**
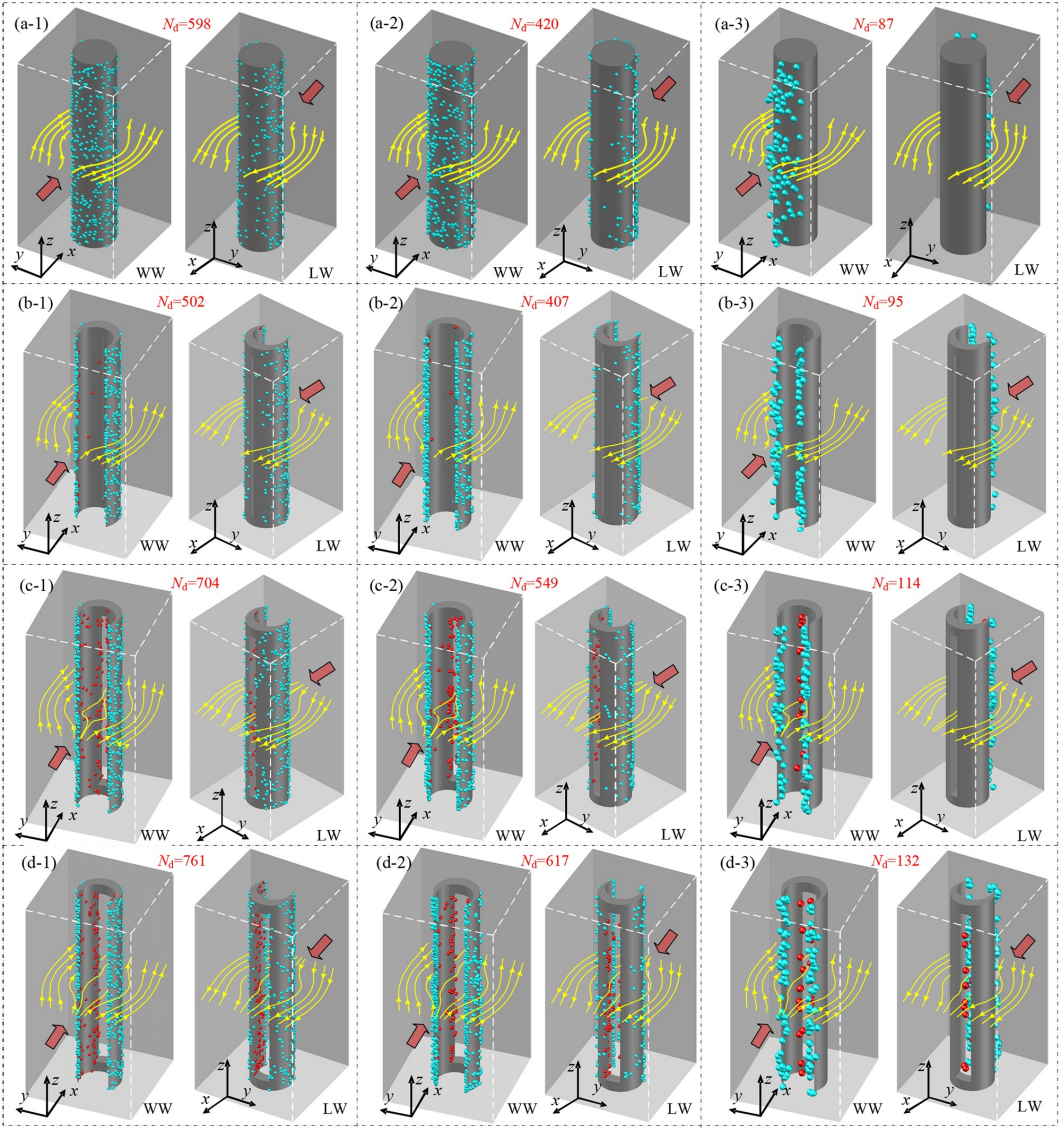
Visualizations of deposited particle distribution on the fiber surface. (a) The circular fiber, (b, c, d) The slit-crescent-shaped fiber with *δ* = 0.05, 0.2, 0.4 respectively when *ε* = 0.15, (a-1, b-1, c-1, d-1) *d*_p_ = 0.1μm, (a-2, b-2, c-2, d-2) *d*_p_ = 0.5μm, (a-3, b-3, c-3, d-3) *d*_p_ = 2.5μm. “WW” represents windward of the fiber, “LW” represents leeward of the fiber, *N*_d_ represents the number of deposited particles, Sky blue spheres represent deposited particles on the outer surface of the fiber, Red spheres represent deposited particles on the inner surface of the fiber.

Particle deposition distributions of the slit-crescent-shaped fiber are displayed in Fig 7 for three particle sizes (*d*_p_ = 0.1μm, 0.5μm, 2.5μm) and three dimensionless slit widths (*δ* = 0.05, 0.2, 0.4). In Fig 7, particle deposition distributions of circular fibers are also included for comparison. Results of particle deposition distribution for *d*_p_ = 0.1μm, 0.5μm and 2.5μm are obtained after 80 000, 150 000 and 3 000 particles have been injected from the inlet of the domain separately. Note that the deposited particles on the surface of the fiber only represent the position of deposited particle, not its true size.

As displayed in Fig 7, for the case of *d*_p_ = 0.1μm and *d*_p_ = 0.5μm where Brownian diffusion play an important role, particles can deposit on any position of the fiber surface and deposited particle number of which decreases gradually from the windward surface of the fiber to the leeward surface, while large particle (*d*_p_ = 2.5μm) dominated by inertia impaction can only deposit on the windward surface of the fiber. It is particularly interesting that there is different spatial distribution of deposited particles between slit-crescent-shaped fibers and circular fibers. Quite a number of particles can go into the slit of the slit-crescent-shaped fiber, which is different from the circular fiber. This can be seen in Fig 7 that the longitudinal split is filled with deposited particles, indicating that the slit-crescent-shaped fiber can capture more particles than circular fiber due to its novel geometry structure.

It should be noted that particles deposited inside the slit of the slit-crescent-shaped fiber will not significantly increase the frontal projected area and consequently keep a lower increase rate in pressure drop. This deposition behavior can alleviate clogging and thus extend the service life of the filter to a certain extent.

In addition, effects of slit width on particle deposition distribution of slit-crescent-shaped fibers are also examined in Fig 7. In the case of *δ* = 0.05 (Fig 7(b)), particles mainly deposit on the outer surface of the slit-crescent-shaped fiber, and deposited particle number is slightly lower than that of the circular fiber when *d*_p_ = 0.1μm, 0.5μm. As dimensionless slit width increases to *δ* = 0.2 (Fig 7(c)) and *δ* = 0.4 (Fig 7(d)), a large number of particles tend to deposit inside the inner cavity, and number of deposited particles for these three particle sizes is higher than that of the circular fiber. This comparison suggests that the slit-crescent-shaped fiber with a large slit width works better in collecting particles.

### 3.4 Pressure drop

**Fig 8:**
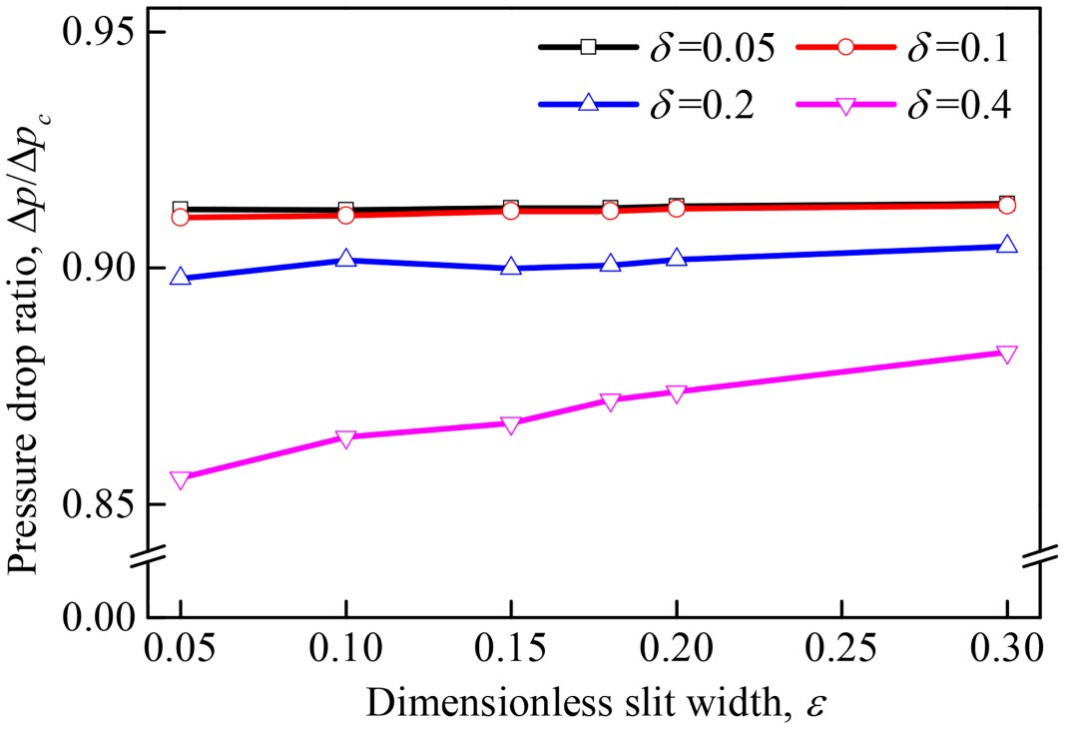
Pressure drop ratios of the slit-crescent-shaped fiber to the circular fiber.

Effects of the dimensionless center-to-center spacing (*ε*) and dimensionless slit width (*δ*) on pressure drop ratios (Δ*p*/Δ*p*_c_) of the slit-crescent-shaped fiber (Δ*p*) to that of the circular fiber (Δ*p*_c_) have been investigated in this section, and results are illustrated in Fig 8.

It can be observed that the pressure drop ratios between the slit-crescent-shaped fiber and the circular fiber are less than 1, indicating that the filtration pressure drop of the former is lower than the later. Moreover, the pressure drop of the slit-crescent-shaped fiber slightly increases as *ε* increases, and decreases as *δ* increases. In fact, for a fixed filtration velocity, pressure drop of the slit-crescent-shaped fiber is determined by both the friction surface area and the windward area, which both depend on *ε* and *δ*. A larger *ε* leads to a larger inner friction surface area, while a larger *δ* leads to a smaller friction surface area and windward area. This means that a slit-crescent-shaped fiber with a small *ε* and large *δ* has much lower pressure drop. For example, when *δ* = 0.4, the filtration pressure drop of the slit-crescent-shaped fiber under various *ε* is reduced by 11.8%–14.4% relative to the circular fiber, which is higher than when *δ* ≤0.2 (8.6%–10.2%).

However, for different *δ* regions, a significant difference in the decrease extent is observed. For a small *δ*, most of the fluid passes around the slit-crescent-shaped fiber, while only a small amount flows through the inner cavity, which means *δ* has a weak influence on the fluid field and pressure drop. This is why there is no significant difference observed for the pressure drop ratio when *δ* increases from 0.05 to 0.1. For a large *δ*, a large amount of fluid flows through the inner cavity, which indicates *δ* has a strong influence on the fluid field on one hand. In addition, enlarged *δ* accompanies with small friction surface area and windward area, which leads to a significant decrease of pressure drop, especially when *δ* is further increased to 0.2 and 0.4.

### 3.5 Collection efficiency

**Fig 9:**
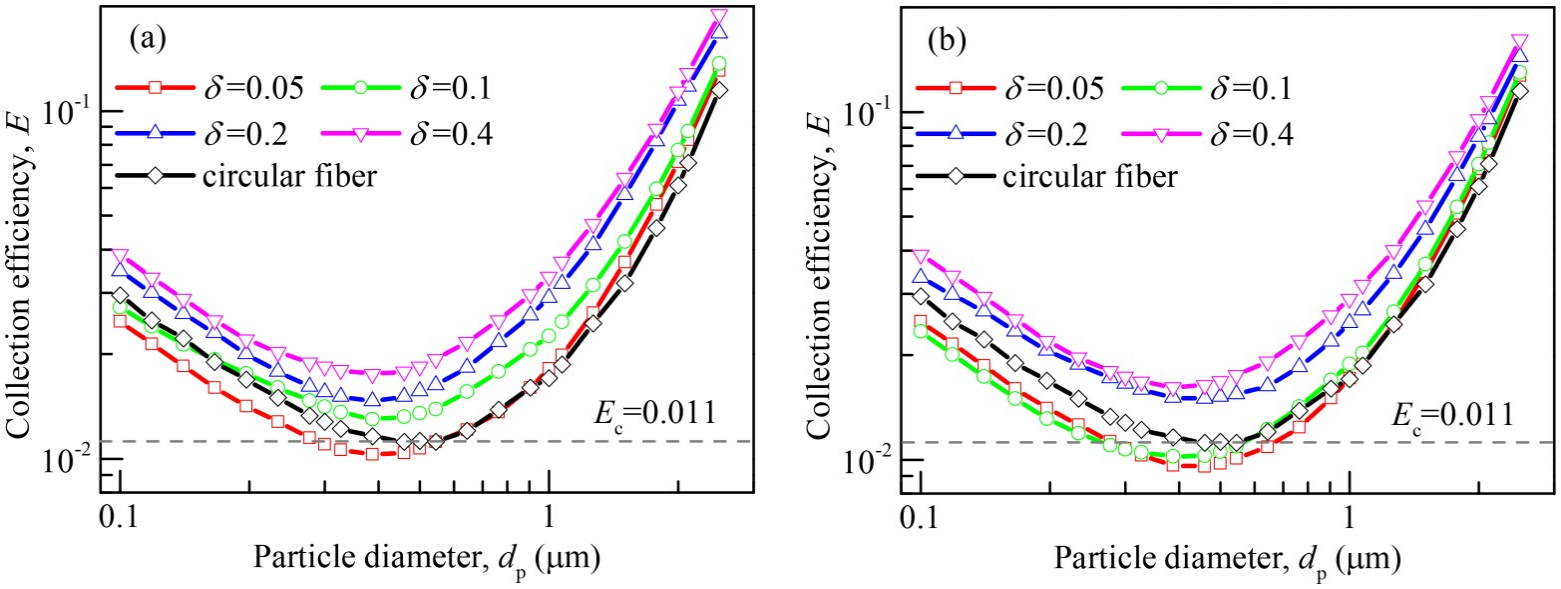
Collection efficiency of the slit-crescent-shaped fiber and the circular fiber. (a) *ε* = 0.05, (b) *ε* = 0.3.

Fig 9 presents variations of collection efficiency (*E*) with the particle diameter at various dimensionless center-to-center spacing (*ε* = 0.05 and 0.3) and dimensionless slit width (*δ* = 0.05, 0.1, 0.2 and 0.4). The collection efficiency of the circular fiber is also given in Fig 9 for comparison. The collection efficiency *E* can be calculated by

$$E=\frac{N_{\text{in}}-N_{\text{out}}}{N_{\text{in}}}\cdot \frac{W}{d_{\text{f1}}}\times 100\%$$ (8)

where *N*_in_ and *N*_out_ are the number of injected particles from inlet of the domain and escaped particles from outlet of the domain, respectively.

As seen in Fig 9, collection efficiency of the slit-crescent-shaped fiber with various *ε* and *δ* has a similar profile as the circular fiber. The variation trend of the collection efficiency with particle size is consistent with that of classical fiber filtration experiments. For small particles where Brownian diffusion plays the most important role in particle capture, all collection efficiency first decreases with the increase of particle diameter, then continues to decrease and reaches a minimum value. Lastly, collection efficiency increases with increasing particle diameter, showing the increasingly enhanced effects of interception and inertia impaction mechanisms.

When *ε* is as small as 0.05, as shown in Fig 9(a), collection efficiency of the slit-crescent-shaped fiber increases with the increase of *δ*. When *δ* = 0.05, the slit-crescent-shaped fiber works much better for particles larger than 1μm, while the circular fiber performs slightly better in dealing with small particles (*d*_p_ <0.5μm). The reasons for this are as follows: (1) particle-laden fluid flowing through the inner cavity of the slit-crescent-shaped fiber is rare due to small slit width, and few particles can enter the slit to deposit, and (2) diffusional deposition depends on the surface area, and the slit-crescent-shaped fiber has a smaller outer deposition surface area than the circular fiber. When *δ* = 0.1, the slit-crescent-shaped fiber has a higher collection efficiency than the circular fiber in most cases, except for when *d*_p_ <0.2μm. As the dimensionless slit width increases to 0.2≤ *δ* ≤0.4, particle laden fluid flowing through the inner cavity of the slit-crescent-shaped fiber increases, and large numbers of particles tend to deposit inside the slit, leading to a rise in collection efficiency.

At the large *ε* of 0.3, as shown in Fig 9(b), the slit-crescent-shaped fiber perform similarly to the situation of *ε* = 0.05. However, collection efficiency for the case of *ε* = 0.3 is lower than that of *ε* = 0.05, especially for *δ* smaller than 0.2 as shown in Fig 9. This is because that the enlarged inner friction surface area accompanies with the large ε, which increases the flow resistance and leads to the decrease of flow rate of particle-laden fluid flowing through the inner cavity of the slit-crescent-shaped fiber, and therefore, less particles can enter the slit to deposit.

In order to quantitatively analyze increased collection efficiency percentage of the slit-crescent-shaped fiber over the circular fiber, the collection efficiency enhancement factor (*λ*) is introduced here, which is defined as follows:

$$\lambda =\frac{E-E_{\text{c}}}{E_{\text{c}}}\times 100\%$$ (9)

where *E* and *E*_c_ are collection efficiency of the slit-crescent-shaped fiber and the circular fiber, respectively.

**Fig 10:**
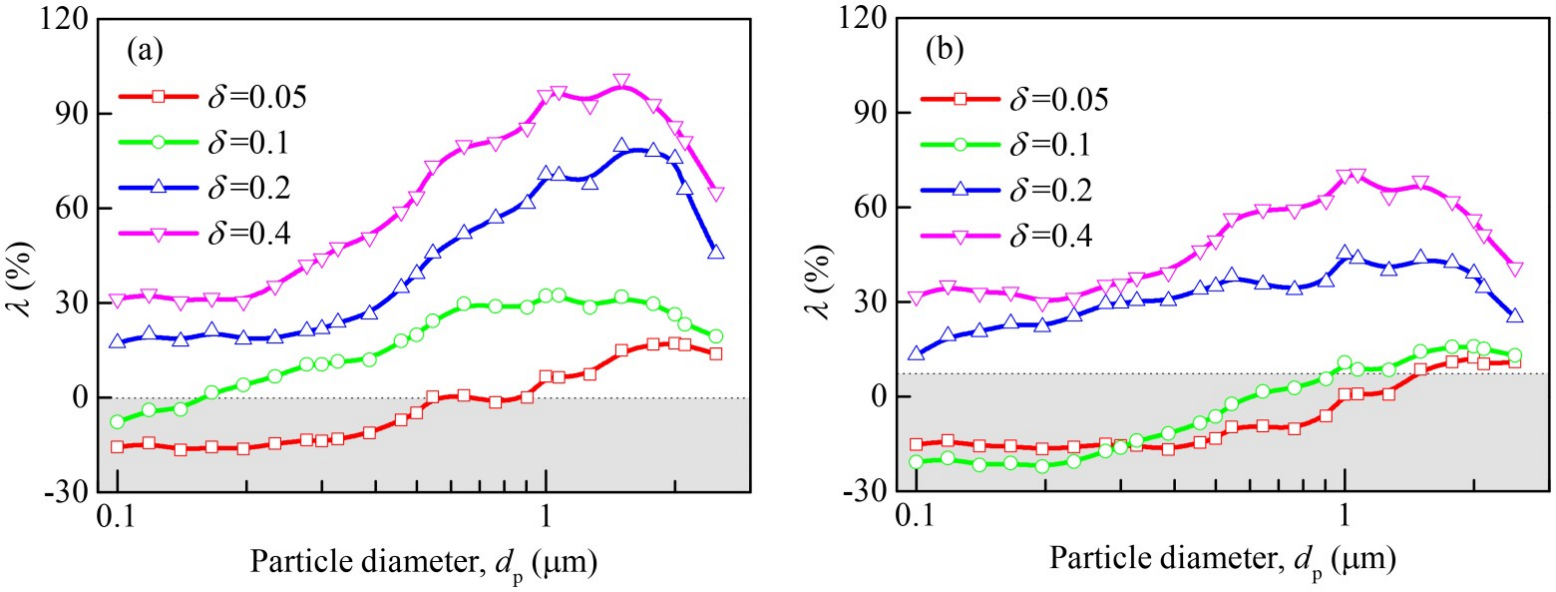
Collection efficiency enhancement factor of the slit-crescent-shaped fiber over the circular fiber. (a) *ε* = 0.05, (b) *ε* = 0.3.

Fig 10 shows the collection efficiency enhancement factor *λ* for the cases with *δ* sets to 0.05, 0.1, 0.2 and 0.4. For a small *ε* (*ε* = 0.05), as illustrated in Fig 10(a), when *δ* = 0.05, the slit-crescent-shaped fiber can enhance filtration of particles larger than 1μm when compared with the circular fiber, and *λ* can arrive at as high as 32%, while for particles smaller than 1μm, however, *λ*≤0, indicating that the circular fiber does much better. When *δ* = 0.1, *λ* is increased when compared with *δ* = 0.05, and generally larger than zero, suggesting that the slit-crescent-shaped fiber is better than the circular fiber for most cases. As dimensionless slit width increases to 0.2≤ *δ* ≤0.4, the slit-crescent-shaped fiber can achieve conspicuous collection efficiency improvement in all cases, and bring about a collection efficiency improvement larger than 17.45% and 31.3% for particles ranging from 0.1μm to 2.5μm when *δ* = 0.2 and *δ* = 0.4 separately. Moreover, compared with small particles (*d*_p_ <0.5μm) and large particle (*d*_p_ = 2.5μm), a much higher collection efficiency enhancement factor for intermediate particles can be found in Fig 10(a), and *λ* even arrives at 101.1% for *d*_p_ = 1.5μm. This is because that the motion trajectories of small particles and large particles are weakly affected by various flow field induced by different fiber cross-sections. Thus, collection efficiency improvement of the slit-crescent-shaped fiber relative to the circular one is relatively weak for these particles.

In the case of a large *ε* (*ε* = 0.3), as shown in Fig 10(b), the slit-crescent-shaped fiber can bring about a collection efficiency improvement larger than 13.1% and 31.8% for all particles when *δ* = 0.2 and *δ* = 0.4 separately, while usually performs worse than the circular fiber for particles smaller than 1μm when *δ* ≤0.1, which is similar to that of *ε* = 0.05. However, *λ* is decreased when compared with *ε* = 0.05, take *δ* = 0.4 and *d*_p_ = 1μm as an example, *λ* = 95.9% when *ε* = 0.05, whereas *λ* falls to 70.3% when *ε* increases to 0.3.

### 3.6 Figure of merit (or quality factor)

**Fig 11:**
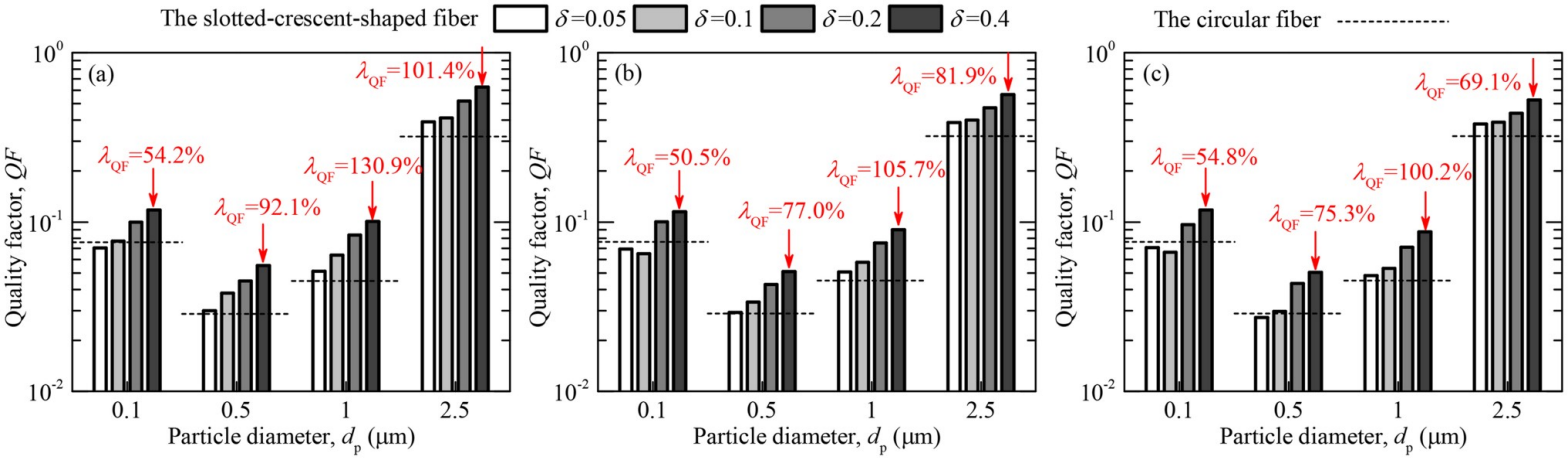
Quality factor of the slit-crescent-shaped fiber and the circular fiber. (a) *ε* = 0.05, (b) *ε* = 0.15, (c) *ε* = 0.3.

Considering four representative particle sizes (*d*_p_ = 0.1μm, 0.5μm, 1μm and 2.5μm) as a case, Fig 11 shows quality factor of the slit-crescent-shaped fiber (*QF*) with various dimensionless center-to-center spacing (*ε*) and dimensionless slit width (*δ*). The calculation results of quality factor for the circular fiber (*QF*_c_) are also shown in Fig 11, which is given by the imaginary horizontal line in the figure. For the sake of argument, the increased quality factor percentage (λ_QF_) of the slit-crescent-shaped fiber over the circular fiber for several representative cases is also given in Fig 11, and λ_QF_ is defined as *λ*_QF_ = (*QF−QF*_c_)/ *QF*_c_×100%.

When *ε* = 0.05, as displayed in Fig 11(a), *QF* of the slit-crescent-shaped fiber increases as *δ* grows, because a larger *δ* leads to a higher collection efficiency and lower pressure drop, which has an advantageous effect on the quality factor. For fine particle (*d*_p_ = 0.1μm), the slit-crescent-shaped fiber has a higher *QF* than the circular fiber when 0.2≤ *δ* ≤0.4, and λ_QF_ reaches up to 54.2%, while it usually performs worse than the circular fiber when *δ* ≤0.1. When collecting intermediate particles (*d*_p_ = 0.5μm, 1μm) and large particle (*d*_p_ = 2.5μm), the slit-crescent-shaped fiber performs better than the circular one, and can bring about 92.1%, 130.9% and 101.4% increase of quality factor respectively for these three particles, which is higher than when *d*_p_ = 0.1μm. This is because the slit-crescent-shaped fiber has a lower collection efficiency for small particles than other particles.

As dimensionless center-to-center spacing increases to *ε* = 0.15 (Fig 11(b)) and *ε* = 0.3 (Fig 11(c)), the variation trend of *QF* with *d*_p_ and *δ* is similar to that of *ε* = 0.05. However, a larger *ε* leads to a lower collection efficiency and higher pressure drop, which results in a lower *QF* than the situation of *ε* = 0.05. Take *d*_p_ = 0.5μm and *δ* = 0.4 as a case study, *λ*_QF_ = 92.1% when *ε* = 0.05, whereas *λ*_QF_ falls to 74.6% and 70.1% when *ε* increases to 0.15 and 0.3.

## 4. Conclusions

In this study, an effective novel non-circular fiber with slit-crescent-shaped cross-section is employed to lower pressure drop and enhance collection efficiency. Effects of fiber structural parameters, including dimensionless center-to-center spacing and dimensionless slit width on filtration performance of the slit-crescent-shaped fiber are investigated by numerical methods. Pressure drop and collection efficiency are compared between the novel fiber and the circular fiber. The results show that the slit-crescent-shaped fiber can efficiently reduce pressure drop by up to 14.4% relative to the circular fiber, and increase collection efficiency. At optimal dimensionless slit width range (0.2≤ *δ* ≤0.4), where the slit-crescent-shaped fiber has a much higher collection efficiency than the circular fiber, and collection efficiency increased percentage relative to the circular fiber varies from 13.1% to 101.1% for particles in the range of 0.1μm–2.5μm. A better comprehensive filtration performance (quality factor) of the slit-crescent-shaped fiber can be achieved by optimizing structural parameters, i.e., a slit-crescent-shaped fiber with a large dimensionless slit width and small dimensionless center-to-center spacing works much better than the circular fiber, especially for particles lager than 0.5μm.

## Supporting information

S1 Raw data (XLSX)

S1 Raw images (PDF)

## References

[1] Yang M, Zhou R, Qiu X, Feng X, Sun J, Wang Q, et al Artificial intelligence-assisted analysis on the association between exposure to ambient fine particulate matter and incidence of arrhythmias in outpatients of Shanghai community hospitals. Environ Int. 2020; 139: 105745 10.1016/j.envint.2020.105745 . 32334122

[2] Xu M, Sbihi H, Pan X, Brauer M. Modifiers of the effect of short-term variation in PM_2.5_ on mortality in Beijing, China. Environ Res. 2020; 183: 109066 10.1016/j.envres.2019.109066 . 32058147

[3] Yang C. Aerosol filtration application using fibrous media-An industrial perspective. Chin J Chem Eng. 2012; 20(1): 1–9. 10.1016/S1004-9541(12)60356-5 . 32288411 PMC7128136

[4] Bortolassi ACC, Nagarajan S, de Araújo Lima B, Guerra VG, Aguiar ML, Huon V, et al Efficient nanoparticles removal and bactericidal action of electrospun nanofibers membranes for air filtration. Mater Sci Eng C Mater Biol Appl. 2019; 102: 718–729. 10.1016/j.msec.2019.04.094 . 31147044

[5] Leung WW, Hung C. Skin effect in nanofiber filtration of submicron aerosols. Sep Purif Technol. 2012; 92: 174–180. 10.1016/j.seppur.2011.02.020

[6] Shou D, Fan J, Ye L, Zhang H, Qian X, Zhang Z. Inverse problem of air filtration of nanoparticles: optimal quality factors of fibrous filters. J Nanomater. 2015; 16(1): 351 10.1155/2015/168392

[7] Przekop R, Gradon L. Deposition and filtration of nanoparticles in the composites of nano- and microsized fibers. Aerosol Sci Technol. 2008; 42(6): 483–493. 10.1080/02786820802187077

[8] Wang J, Kim SC, Pui DYH. Figure of merit of composite filters with micrometer and nanometer fibers. Aerosol Sci Technol. 2008; 42(9): 722–728. 10.1080/02786820802249133

[9] Li W, Shen S, Li H. Study and optimization of the filtration performance of multi–fiber filter. Adv Powder Technol. 2016; 27(2): 638–645. 10.1016/j.apt.2016.02.018

[10] Rabiee MB, Talebi S, Abouali O, Izadpanah E. Investigation of the characteristics of particulate flows through fibrous filters using the lattice Boltzmann method. Particuology. 2015; 21(4): 90–98. 10.1016/j.partic.2014.11.010

[11] Yun KM, Suryamas AB, Iskandar F, Bao L, Niinuma H, Okuyama K. Morphology optimization of polymer nanofiber for applications in aerosol particle filtration. Sep Purif Technol. 2010; 75(3): 340–345. 10.1016/j.seppur.2010.09.002

[12] Wang N, Si Y, Wang N, Sun G, Elnewehy MH, Aldeyab SS, et al Multilevel structured polyacrylonitrile/silica nanofibrous membranes for high-performance air filtration. Sep Purif Technol. 2014; 126(15): 44–51. 10.1016/j.seppur.2014.02.017

[13] Viswanathan G, Kane DB, Lipowicz PJ. High efficiency fine particulate filtration using carbon nanotube coatings. Adv Mater. 2004; 16(22): 2045–2049. 10.1002/adma.200400463

[14] Sanchez JR, Rodriguez JM, Alvaro A, Estevez AM. The capture of fly ash particles using circular and noncircular cross-section fabric filters. Environ Prog. 2007; 26(1): 50–58. 10.1002/ep.10186

[15] Inagaki M, Sakai K, Namiki N, Emi H, Otani Y. Influence of fiber cross-sectional shape on filter collection performance. Kagaku Kogaku Ronbun. 2001; 27(1): 113–120. 10.1252/kakoronbunshu.27.113

[16] Gu C, Lu S, Li R, Lu D, Wu W, Yuan Z. Influence of fiber on filtration performance for PM_2.5_. CIESC Journal. 2014; 65(6): 2137–2147. 10.3969/j.issn.0438-1157.2014.06.026

[17] Zhu C, Lin C, Cheung CS. Inertial impaction-dominated fibrous filtration with rectangular or circular fibers. Powder Technol. 2000; 112(1–2): 149–162. 10.1016/S0032-5910(99)00315-0

[18] Chen SX, Cheung CS, Chan CK, Zhu C. Numerical simulation of aerosol collection in filters with staggered parallel rectangular fibres. Comput Mech. 2002; 28(2): 152–161. 10.1007/s00466-001-0289-4

[19] Huang H, Zhao H. Numerical study of pressure drop and diffusional collection efficiency of rectangular fibers in filtration. Journal of University of Chinese Academy of Sciences. 2017; 34(2): 210–217. 10.7523/j.issn.2095-6134.2017.02.014

[20] Hosseini SA, Tafreshi HV. On the importance of fibers' cross-sectional shape for air filters operating in the slip flow regime. Powder Technol. 2011; 212(3): 425–431. 10.1016/j.powtec.2011.06.025

[21] Raynor PC. Flow field and drag for elliptical filter fibers. Aerosol Sci Technol. 2002; 36(12): 1118–1127. 10.1080/02786820290092159

[22] Wang J, Pui DYH. Filtration of aerosol particles by elliptical fibers: A numerical study. J Nanopart Res. 2009; 11(1): 185–196. 10.1007/s11051-008-9422-z

[23] Wang W, Xie M, Wang L. An exact solution of interception efficiency over an elliptical fiber collector. Aerosol Sci Technol. 2012; 46(8): 843–851. 10.1080/02786826.2012.671559

[24] Wang H, Zhao H, Wang K, Zheng C. Simulating and modeling particulate removal processes by elliptical fibers. Aerosol Sci Technol. 2014; 48(2): 207–218. 10.1080/02786826.2013.868595

[25] Lin KC, Patel R, Tsai JS. Filtration of aerosol particles by clean elliptical fibers with relevance to pore size: A lattice Boltzmann-cellular automaton model. Comput Fluids. 2017; 156: 534–544. 10.1016/j.compfluid.2017.08.011

[26] Zhu H, Yang H, Fu H, Kang Y. Numerical analysis of filtration pressure drop and inertial collection efficiency for elliptical fibers. China Environmental Science. 2019; 39(2): 565–573. 10.19674/j.cnki.issn1000-6923.2019.0068

[27] Jin X, Yang L, Du X, Yang Y. Modeling filtration performance of elliptical fibers with random distributions. Adv Powder Technol. 2017; 28(4): 1193–1201. 10.1016/j.apt.2017.02.005

[28] Fotovati S, Tafreshi HV, Pourdeyhimi B. Analytical expressions for predicting performance of aerosol filtration media made up of trilobal fibers. J Hazard Mater. 2011; 186(2–3): 1503–1512. 10.1016/j.jhazmat.2010.12.027 . 21216526

[29] Wang K, Zhao H. The influence of fiber geometry and orientation angle on filtration performance. Aerosol Sci Technol. 2015; 49(2): 75–85. 10.1080/02786826.2014.1003278

[30] Munson BR, Okiishi TH, Huebsch WW, Rothmayer AP. Fundamentals of fluid mechanics. 7th ed New York: Wiley; 2012.

[31] Hosseinalipour SM, Namazi M. Pore-scale numerical study of flow and conduction heat transfer in fibrous porous media. J Mech Sci Technol. 2019; 33(5): 2307–2317. 10.1007/s12206-018-1231-4

[32] Crowe CT, Schwarzkopf JD, Sommerfeld M, Tsuji Y. Multiphase flows with droplets and particles. 2nd ed Boca Raton: CRC Press; 2011.

[33] Bodnár T, Galdi GP, Nečasová Š. Particles in Flows Adv Math Fluid Mech. Cham: Birkhäuser/Springer; 2017.

[34] Davies C. Air filtration. New York: Academic Press; 1973.

[35] Li A, Ahmadi G. Dispersion and deposition of spherical particles from point sources in a turbulent channel flow. Aerosol Sci Technol. 1992; 16(4), 209–226. 10.1080/02786829208959550

[36] Longest PW, Xi J. Effectiveness of direct Lagrangian tracking models for simulating nanoparticle deposition in the upper airways. Aerosol Sci Technol. 2007; 41(4): 380–397. 10.1080/02786820701203223

[37] Inthavong K, Tian L, Tu J. Lagrangian particle modelling of spherical nanoparticle dispersion and deposition in confined flows. J Aerosol Sci. 2016; 96: 56–68. 10.1016/j.jaerosci.2016.02.010

[38] Lee KW, Liu BYH. Experimental study of aerosol filtration by fibrous filters. Aerosol Sci Technol. 1981; 1(1): 35–46. 10.1080/02786828208958577

