# Supplementary material for: Numerical calculation and analysis of filtration performance of an effective novel structural fiber for PM2.5
Source: PLoS One. 2020 Oct 22;15(10):e0240941. doi: 10.1371/journal.pone.0240941 (PMC7580886; doi:10.1371/journal.pone.0240941)
Supplement: S1 Raw images — (PDF) [file pone.0240941.s002.pdf]

Raw images

Fig 6. Raw images.

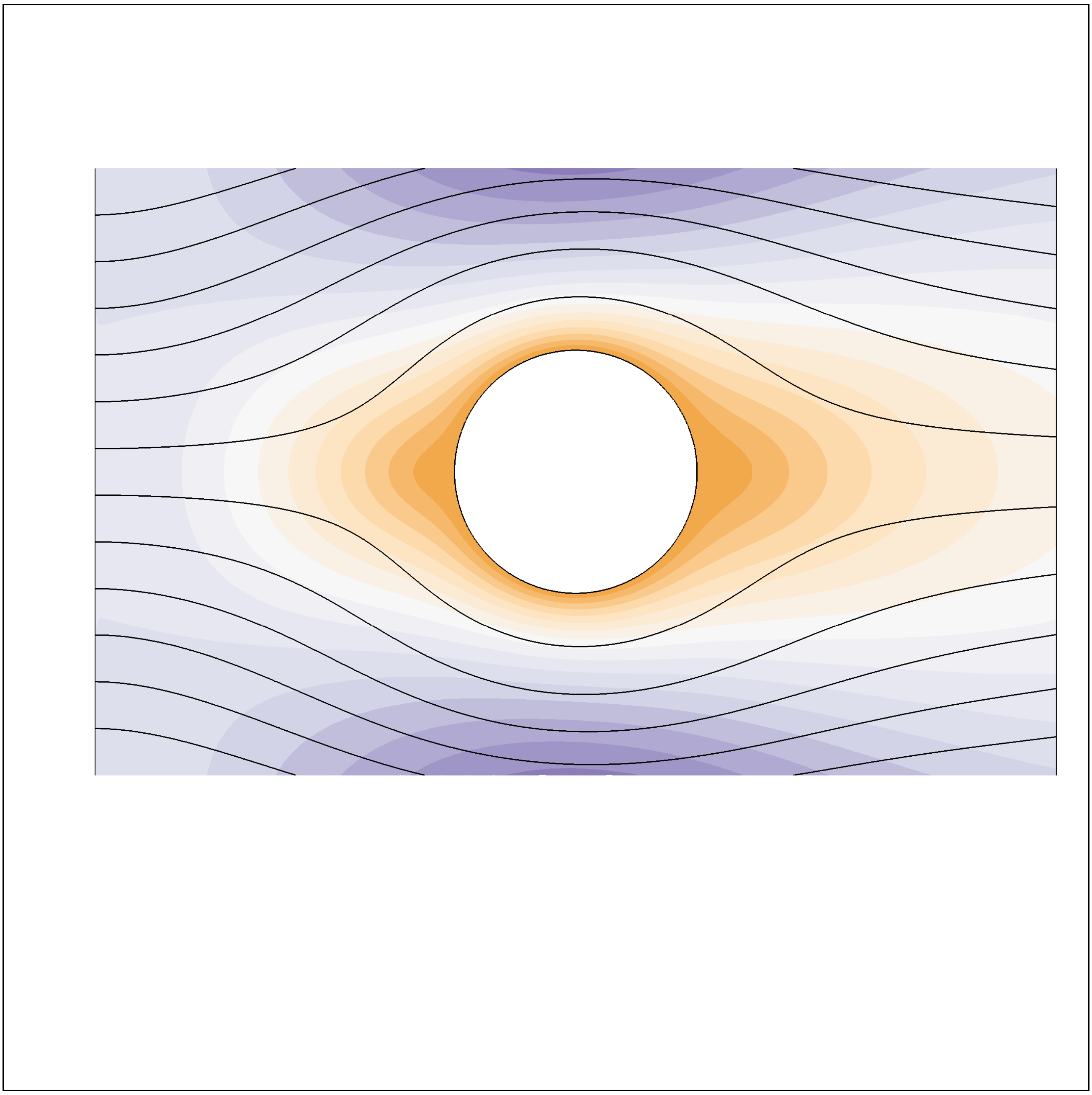

Fig 6-01. Flow Field around the circular fiber

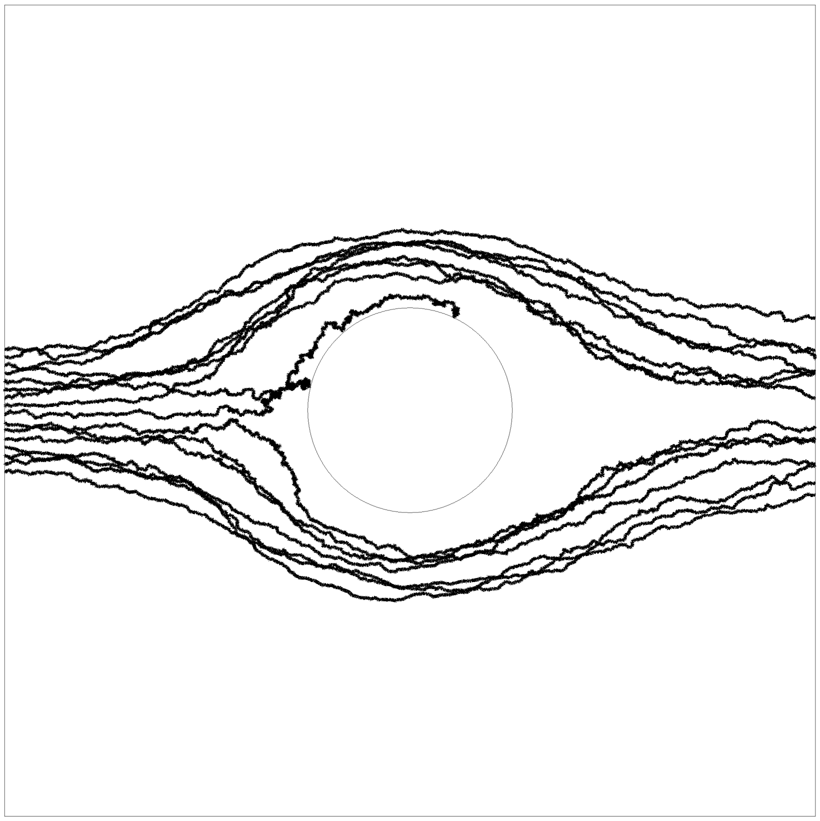

Fig 6-02. Particle trajectories for $d_p = 0.1$ μm.

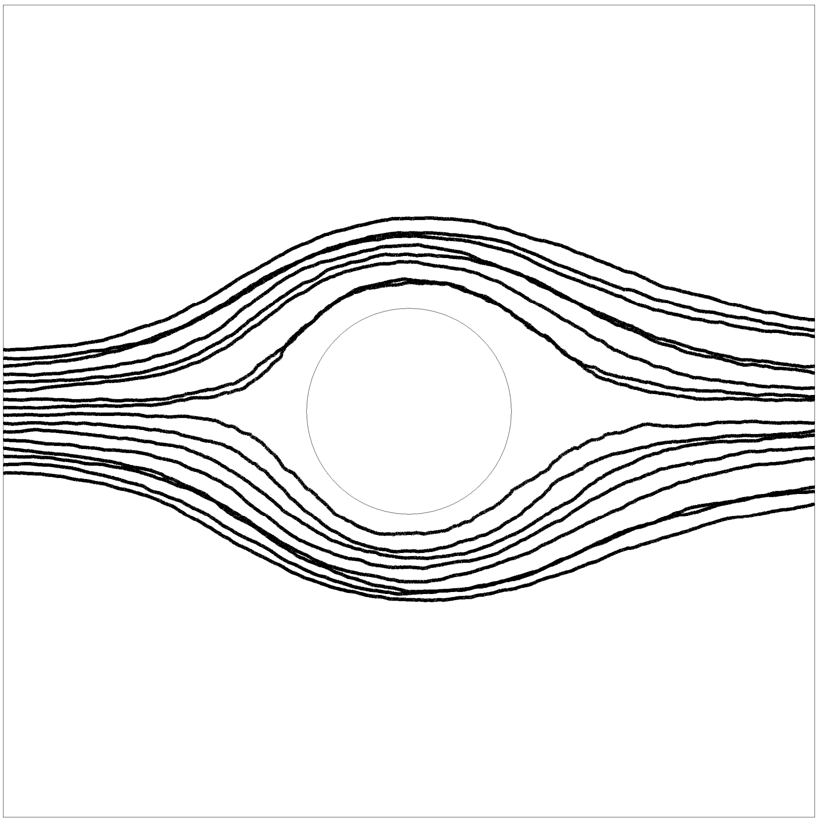

Fig 6-03. Particle trajectories for $d_p = 0.5$ μm.

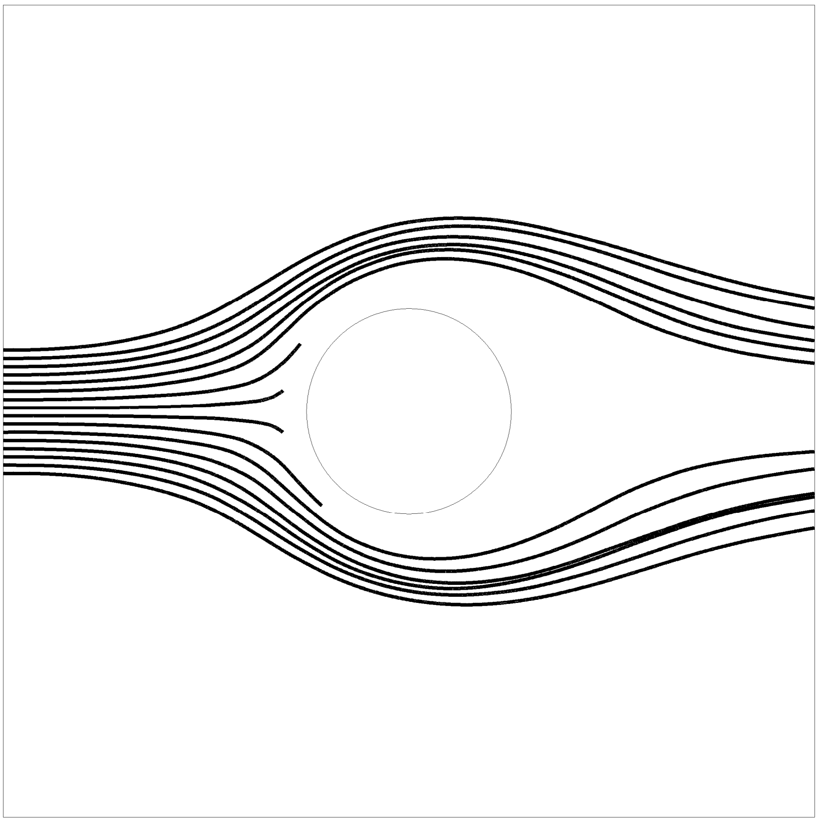

Fig 6-04. Particle trajecto01ries for $d_p = 2.5$ μm.

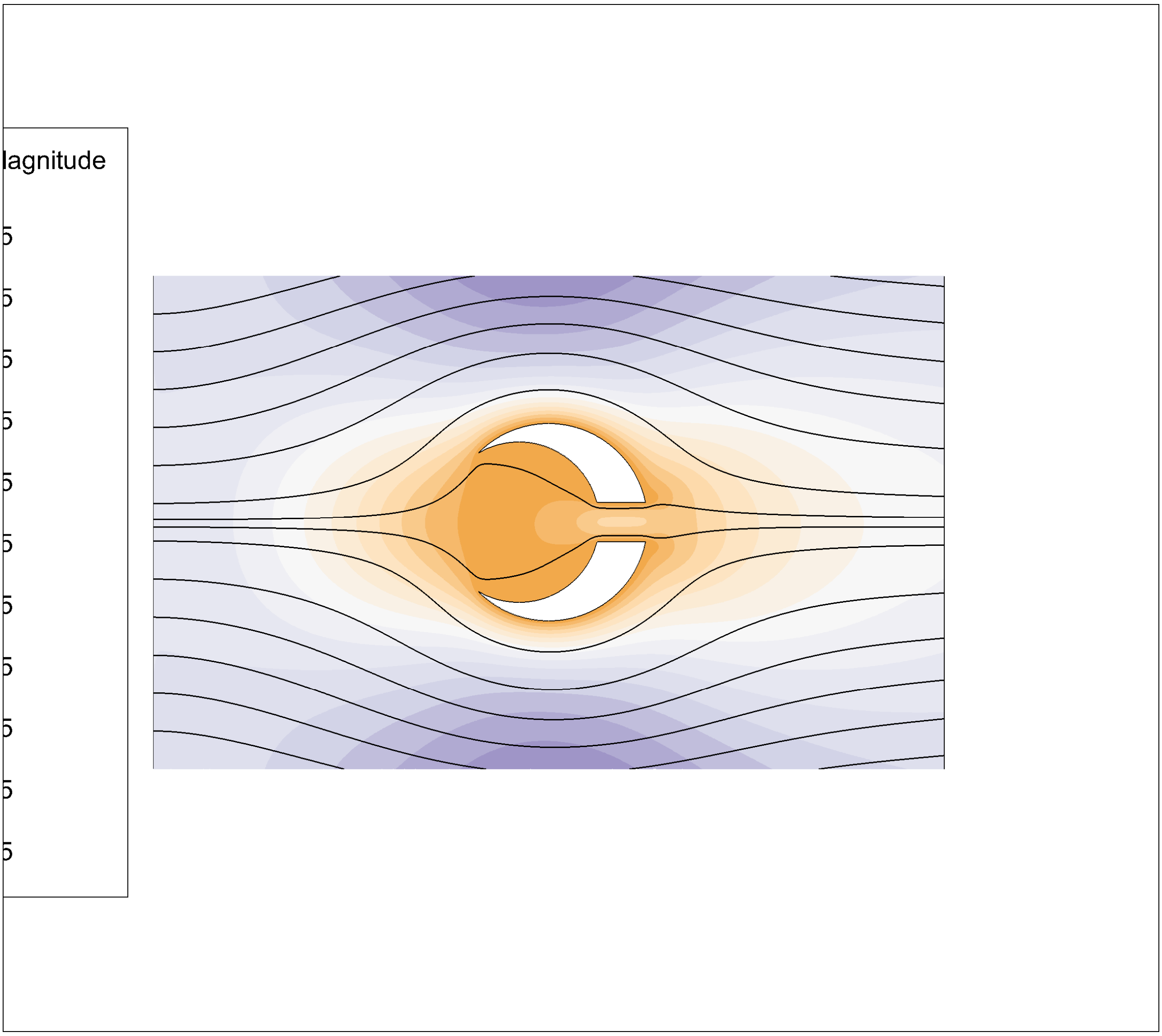

Fig 6-05. Flow field around the slit-crescent-shaped fiber with ε =0.15, δ =0.2.

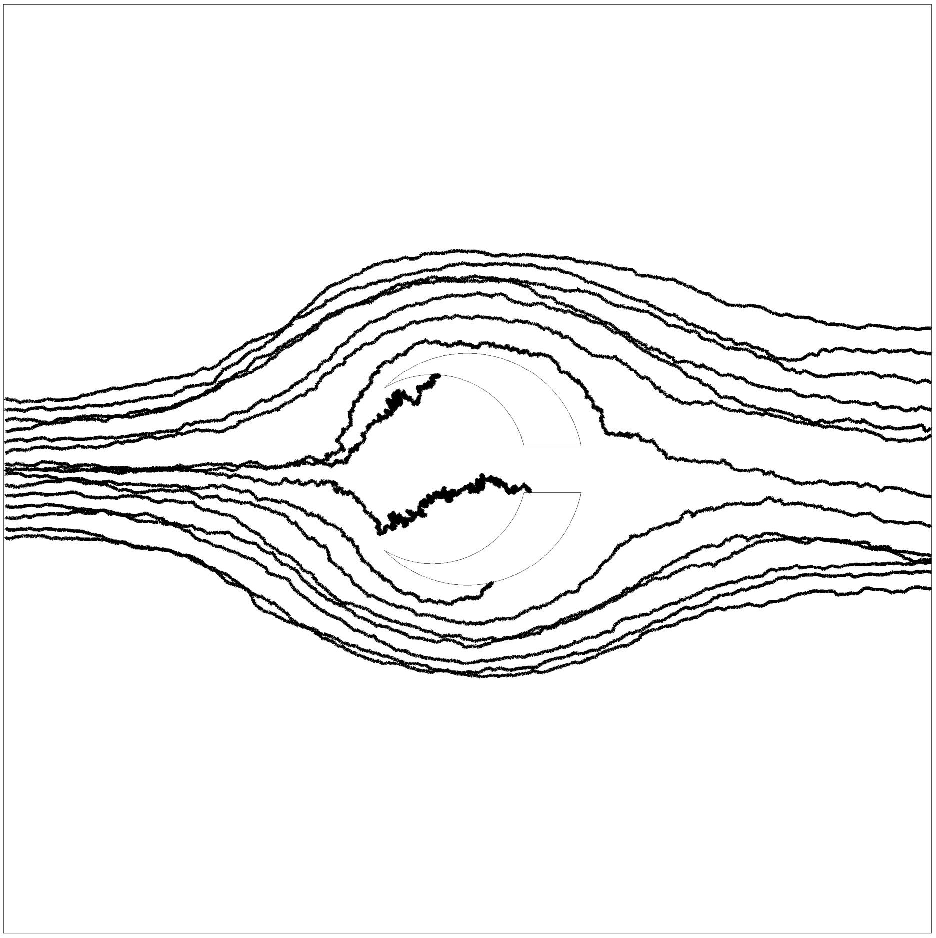

Fig 6-06. Particle trajectories for $d_p = 0.1$ μm.

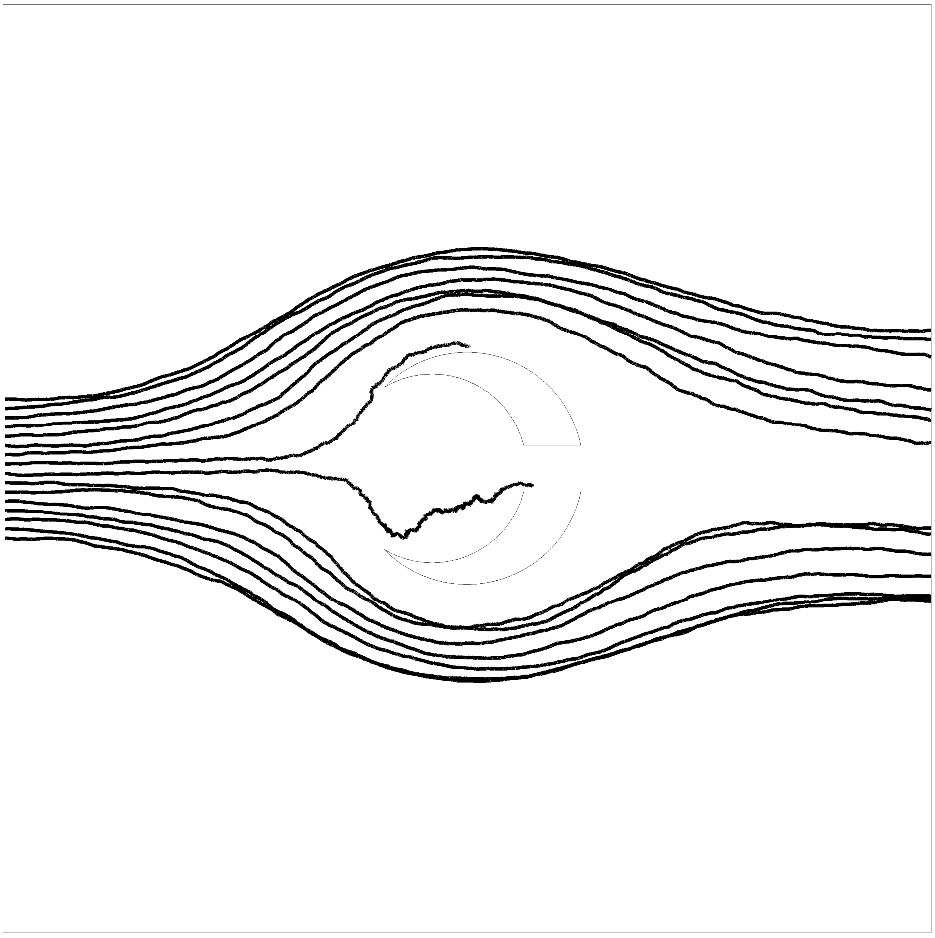

Fig 6-07. Particle trajectories for $d_p = 0.5$ μm.

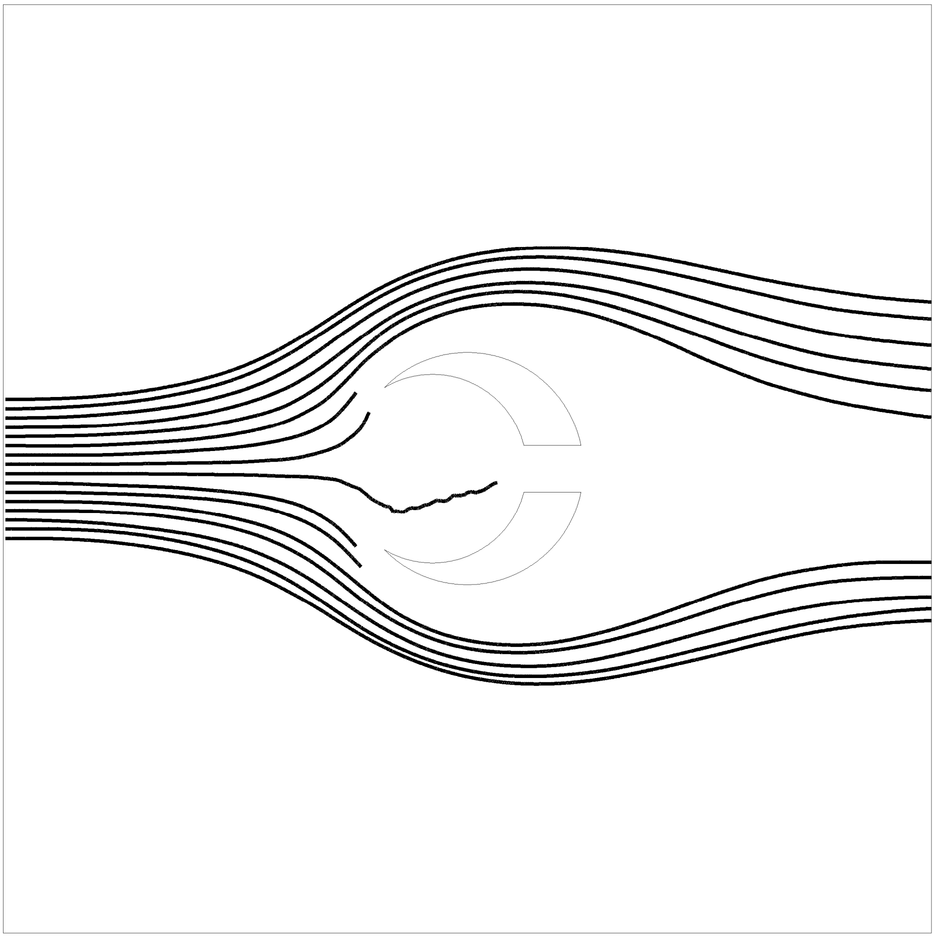

Fig 6-08. Particle trajectories for $d_p = 2.5$ μm.

Fig 7. Raw images.

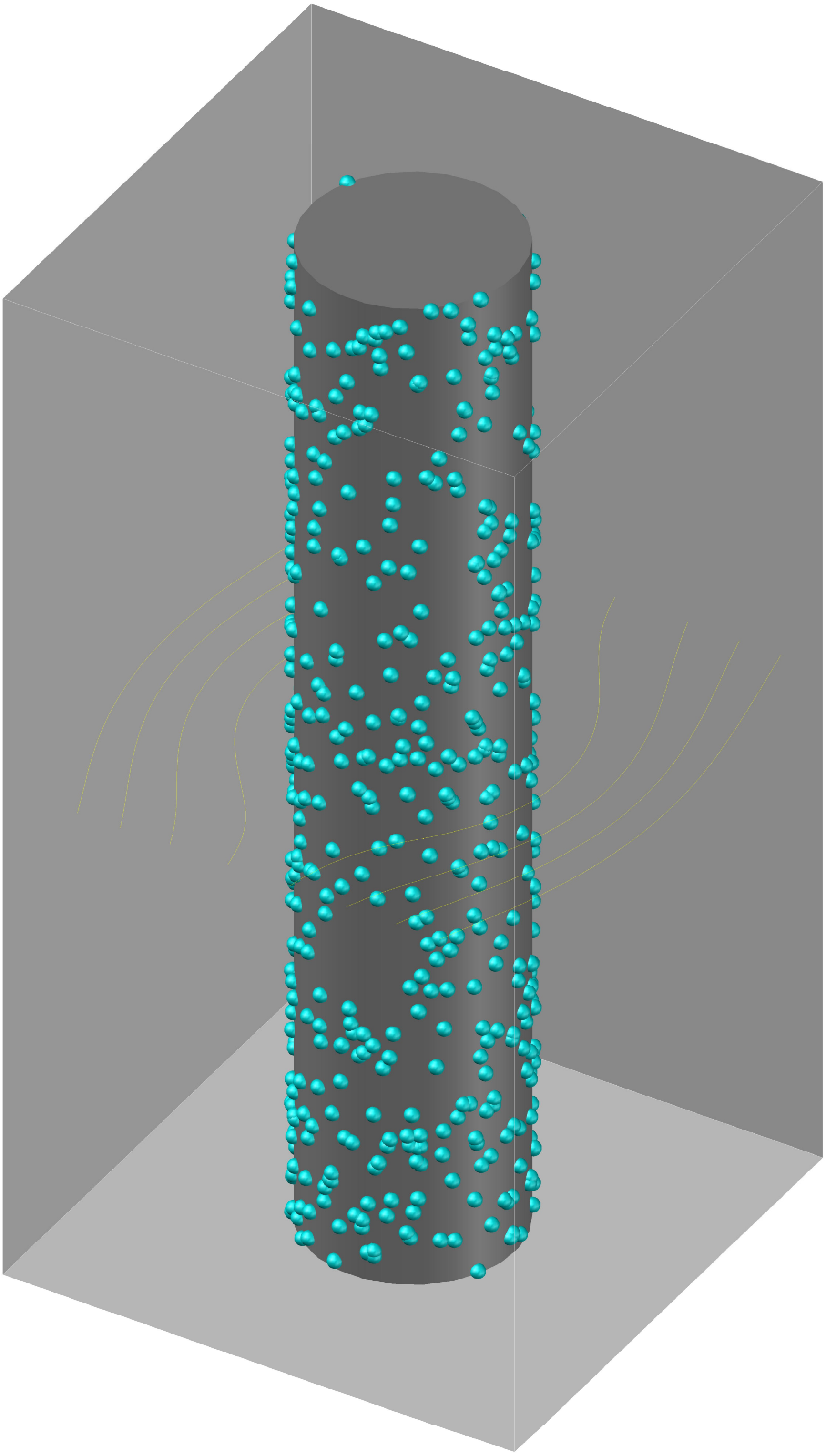

Fig 7-01. Visualizations of deposited particle distribution on the windward of the circular fiber with $d_p = 0.1 \mu m$.

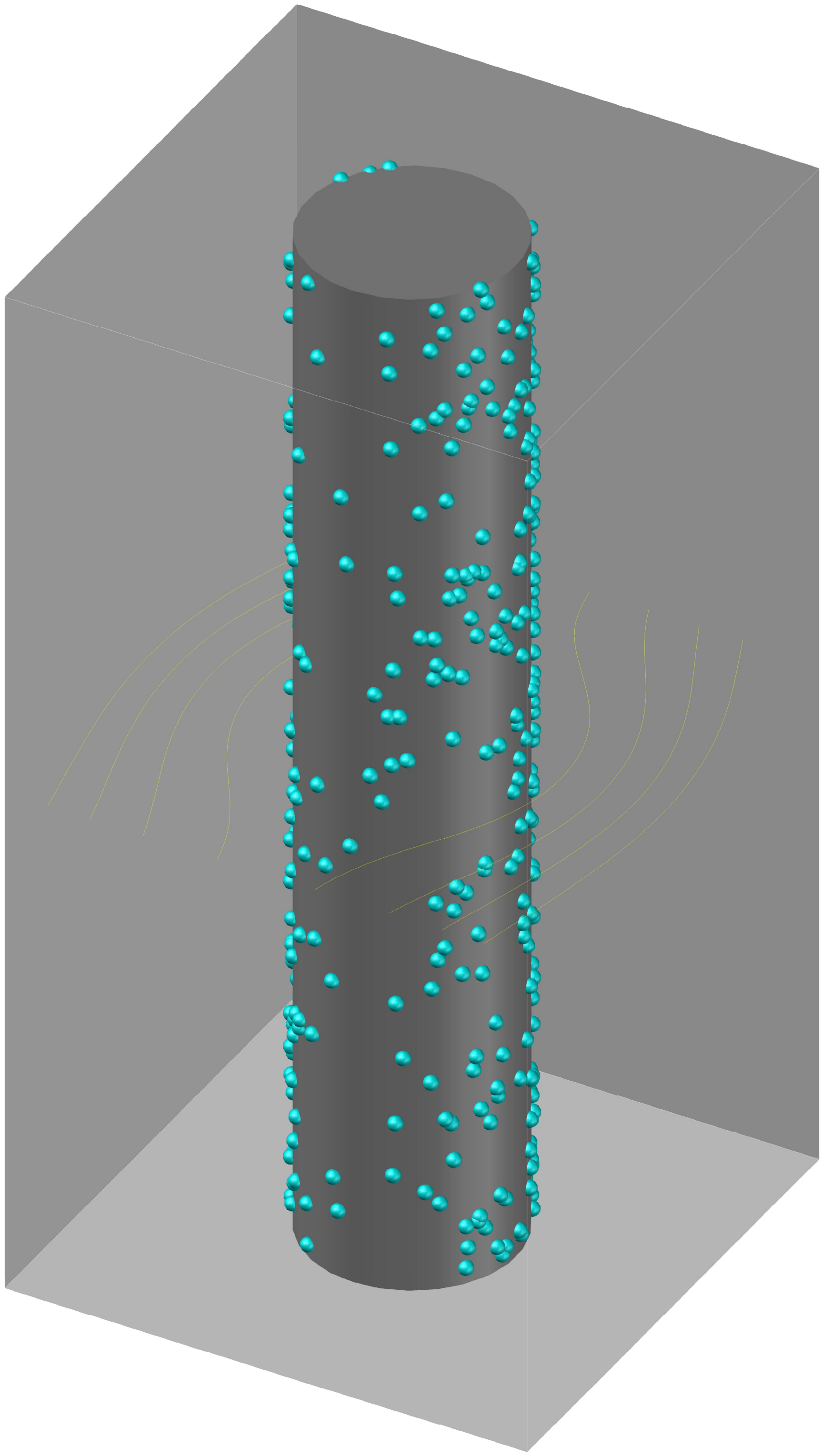

Fig 7-02. Visualizations of deposited particle distribution on the leeward of the circular fiber with d_p = 0.1μm.

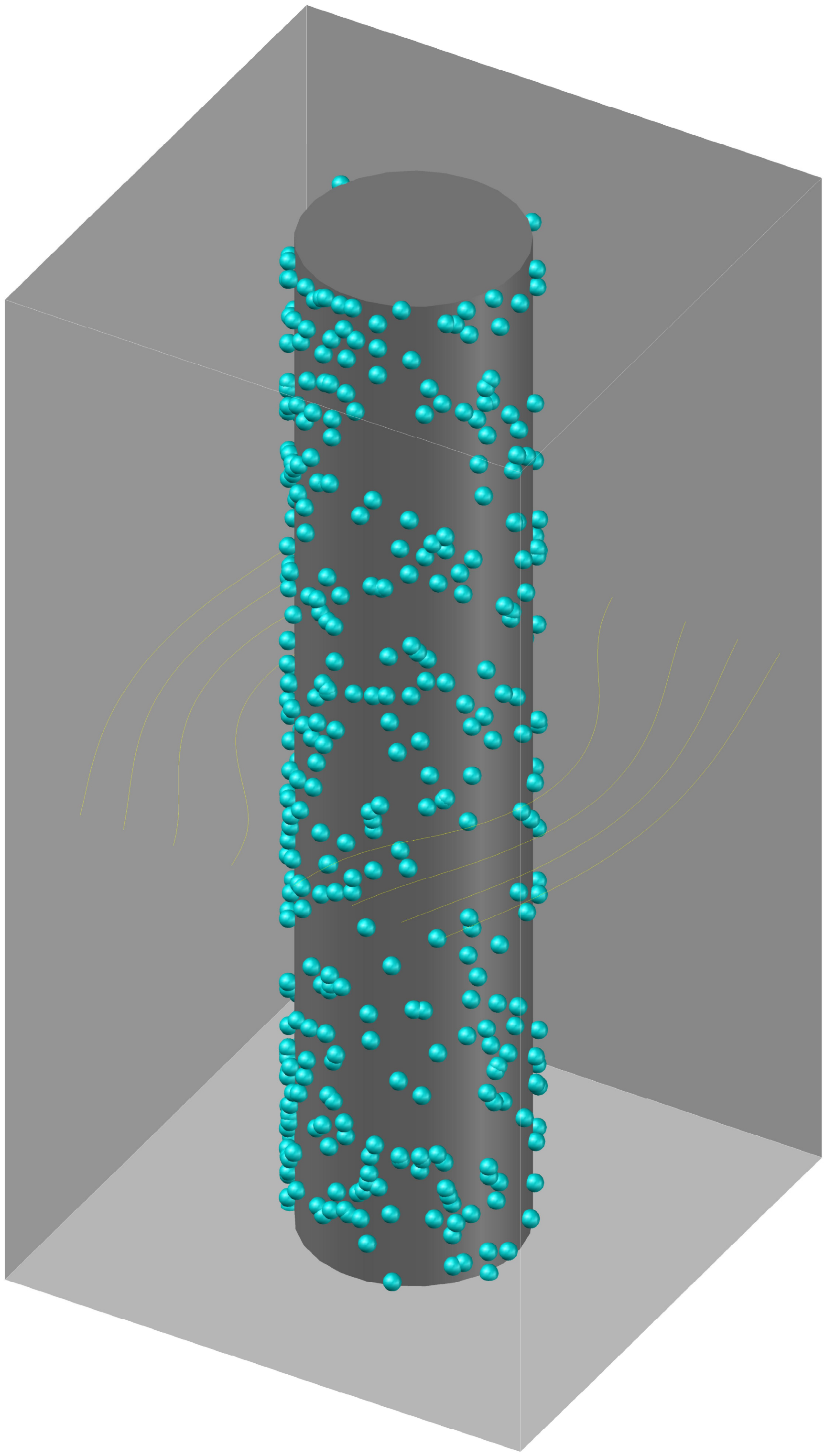

Fig 7-03. Visualizations of deposited particle distribution on the windward of the circular fiber with $d_p = 0.5\mu m$.

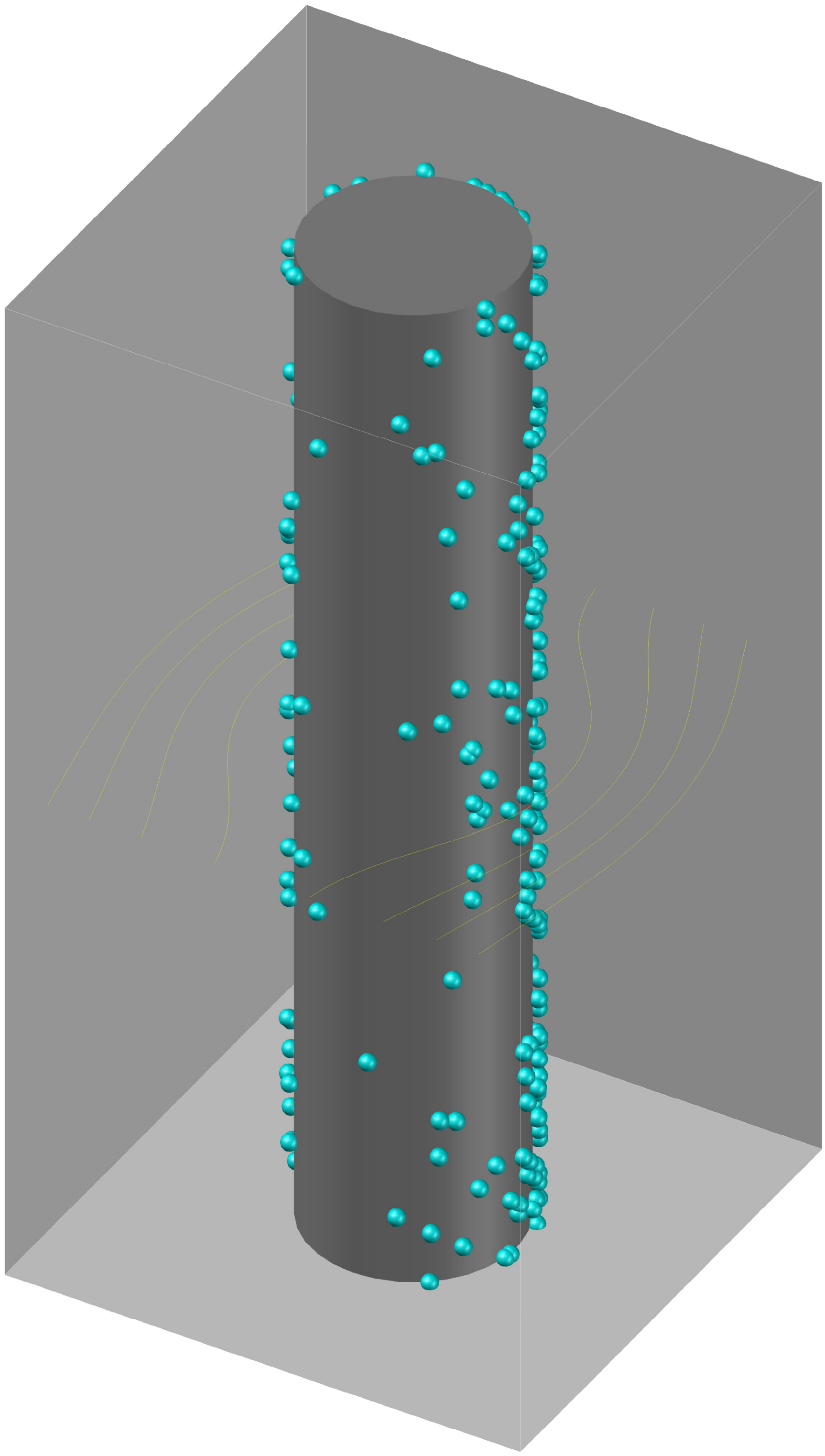

Fig 7-04. Visualizations of deposited particle distribution on the leeward of the circular fiber with d_p = 0.5μm.

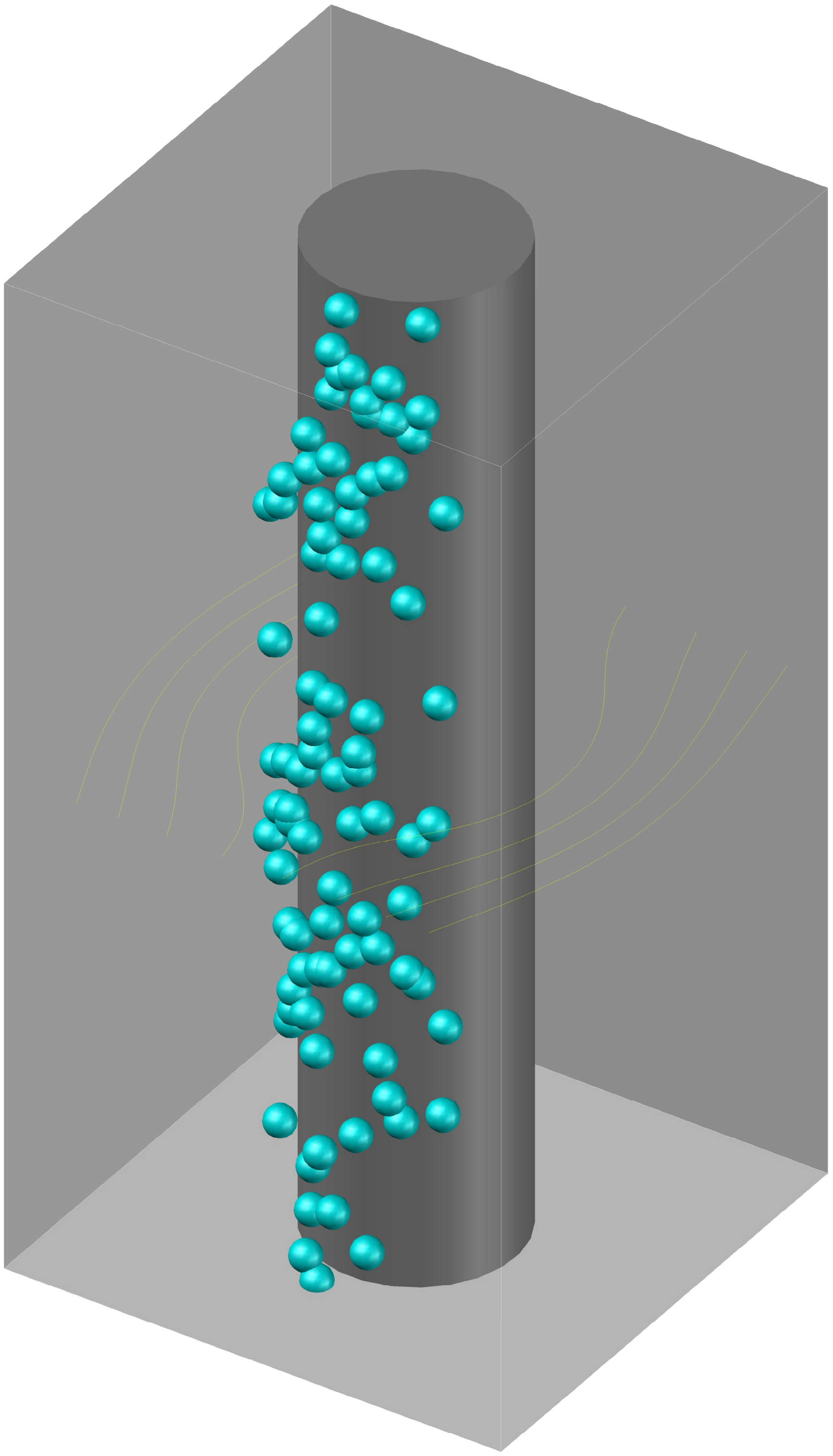

Fig 7-05. Visualizations of deposited particle distribution on the windward of the circular fiber with $d_p = 2.5\mu m$.

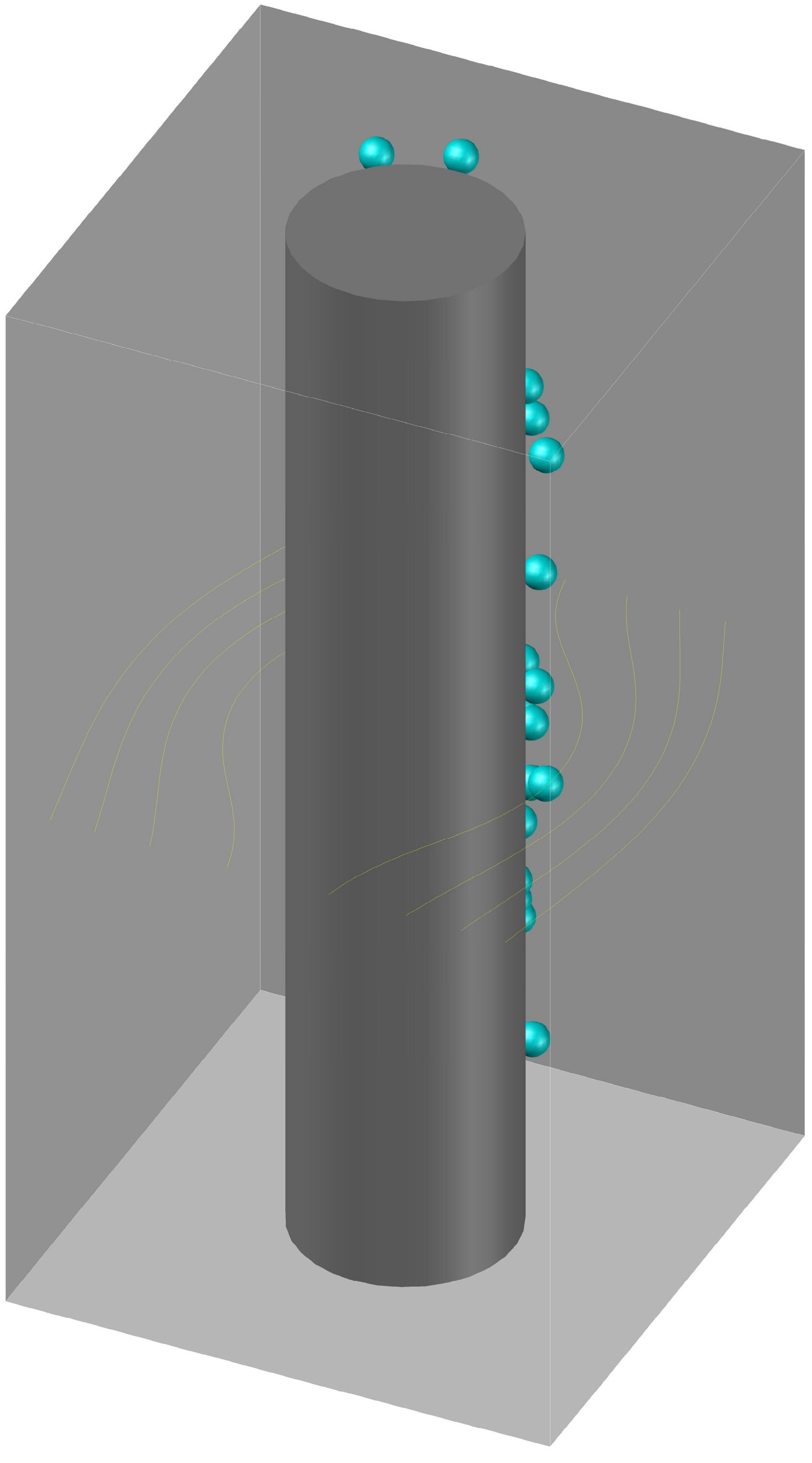

Fig 7-06. Visualizations of deposited particle distribution on the leeward of the circular fiber with d_p = 2.5μm.

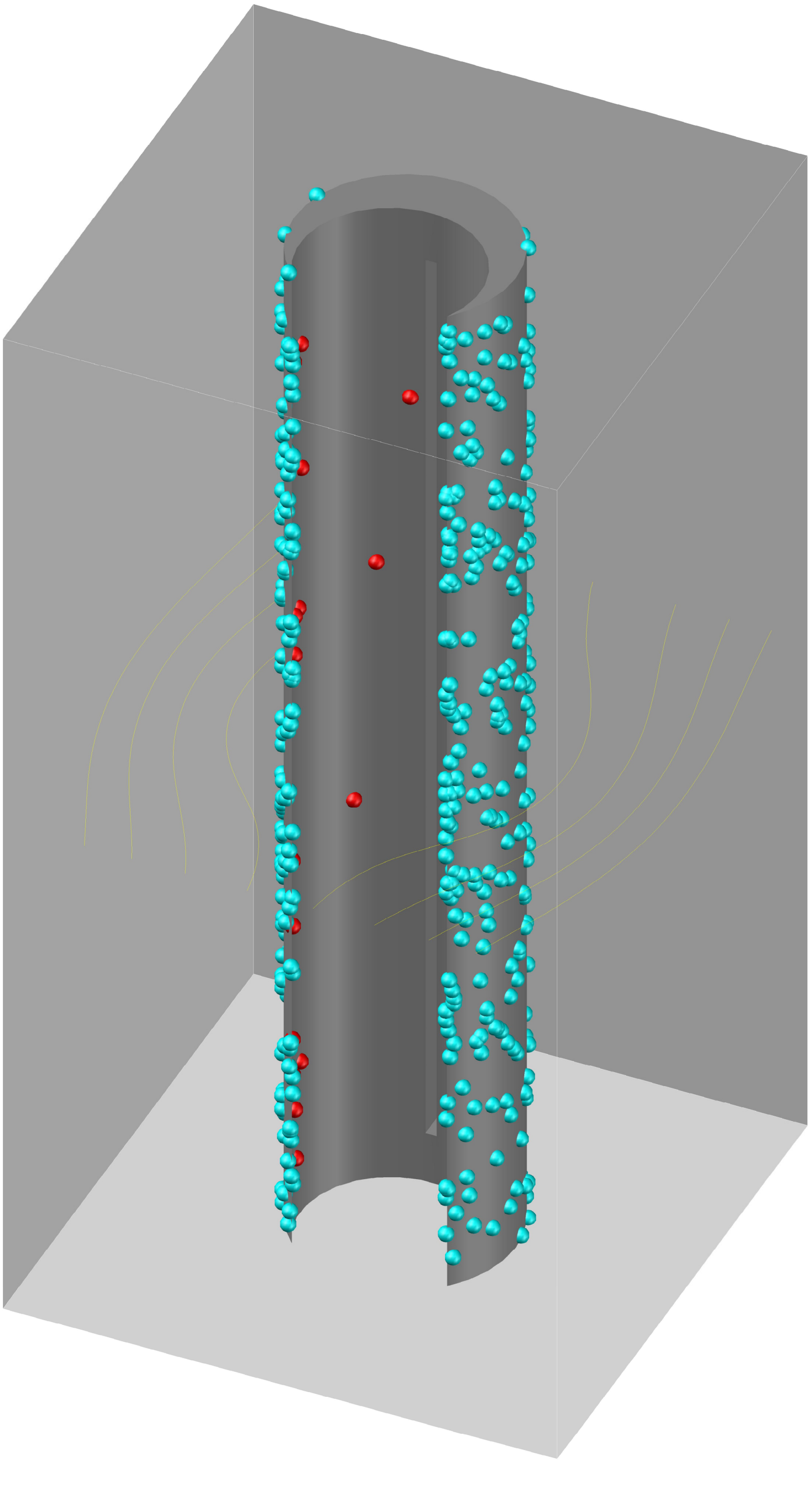

Fig 7-07. Visualizations of deposited particle distribution on the windward of the slit-crescent-shaped fiber with $\delta = 0.05$ and $d_p = 0.1\mu m$.

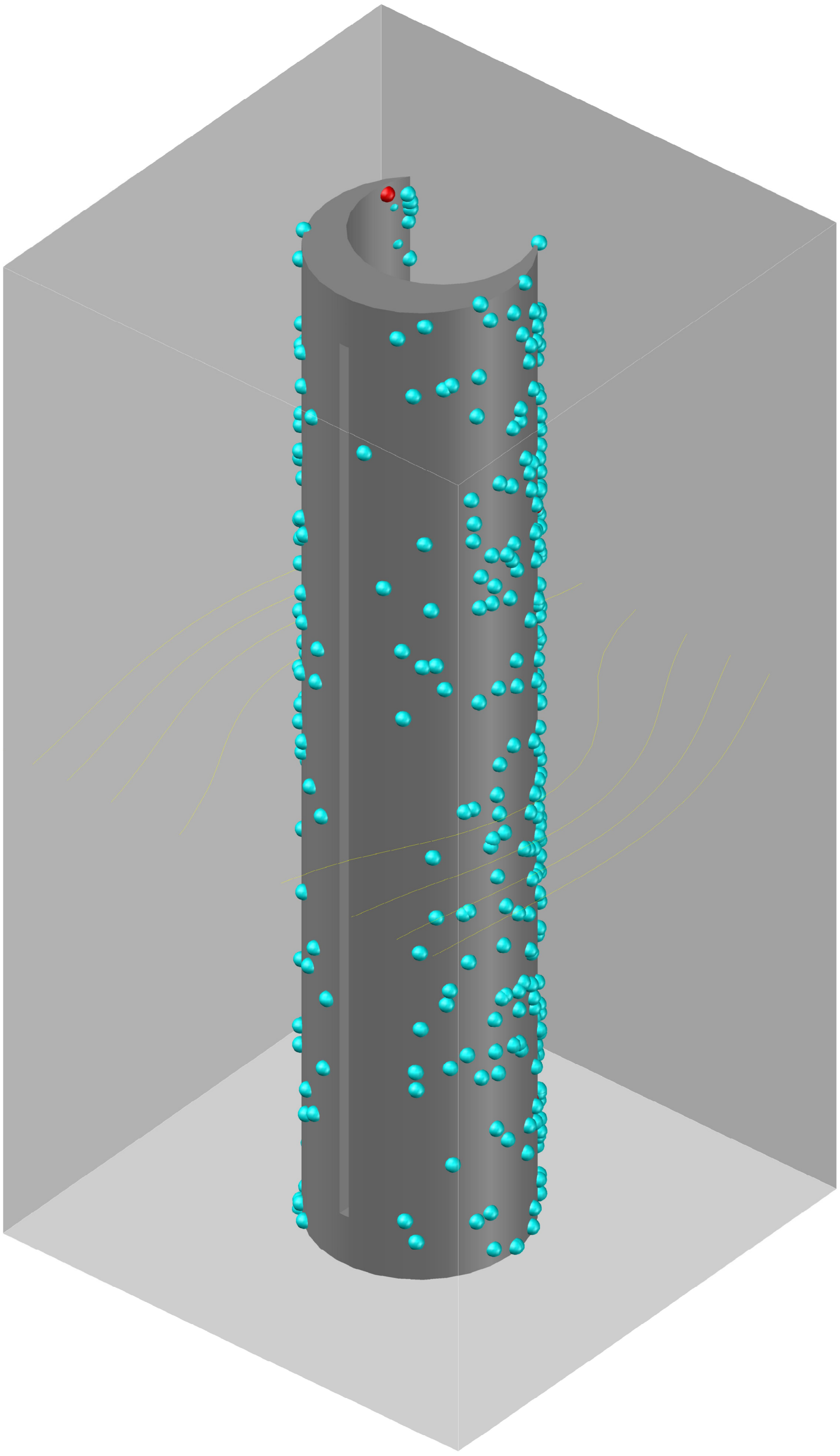

Fig 7-08. Visualizations of deposited particle distribution on the leeward of the slit-crescent-shaped fiber with $\delta = 0.05$ and $d_p = 0.1\mu m$.

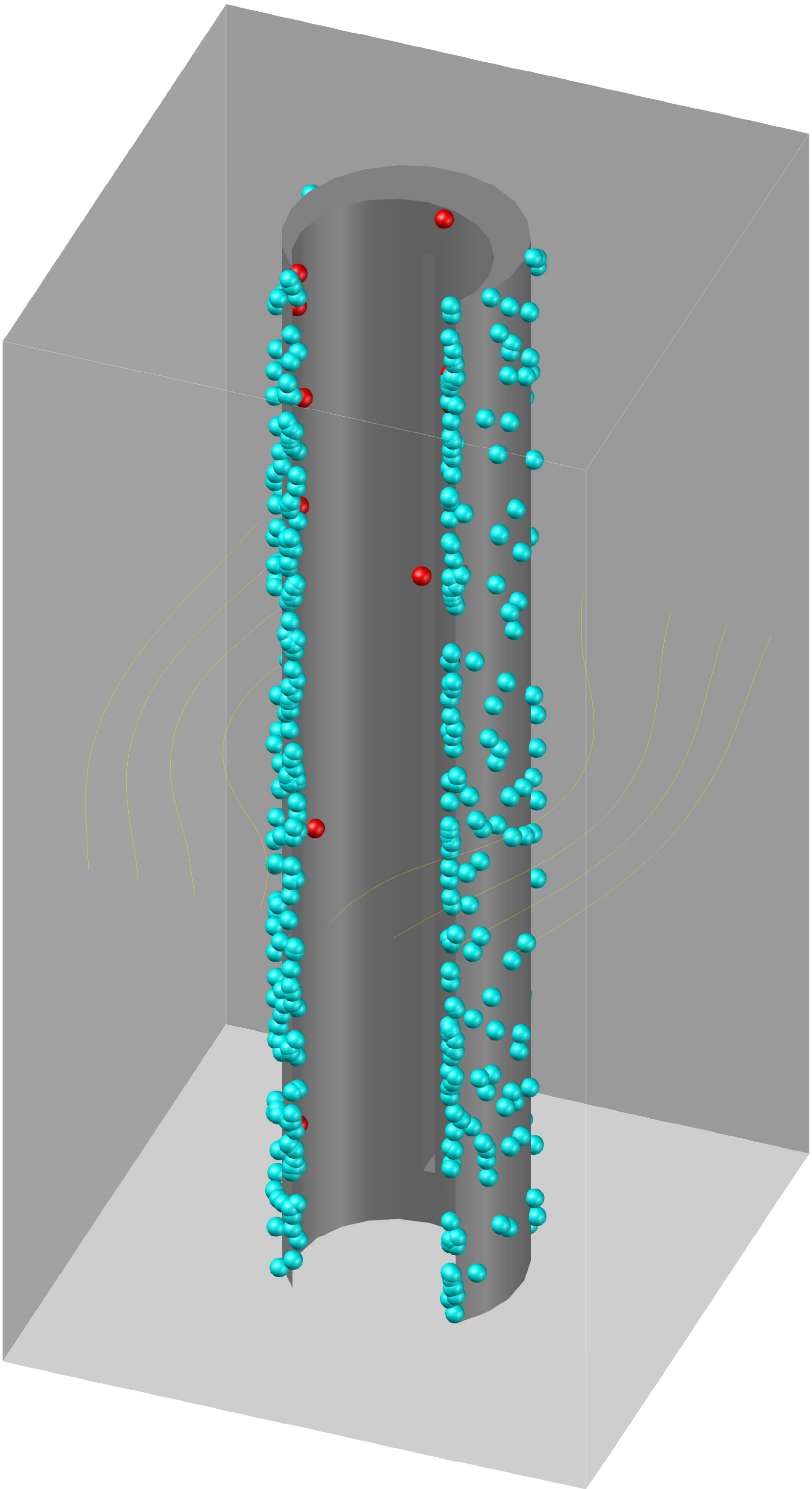

Fig 7-09. Visualizations of deposited particle distribution on the windward of the slit-crescent-shaped fiber with $\delta = 0.05$ and $d_p = 0.5\mu m$.

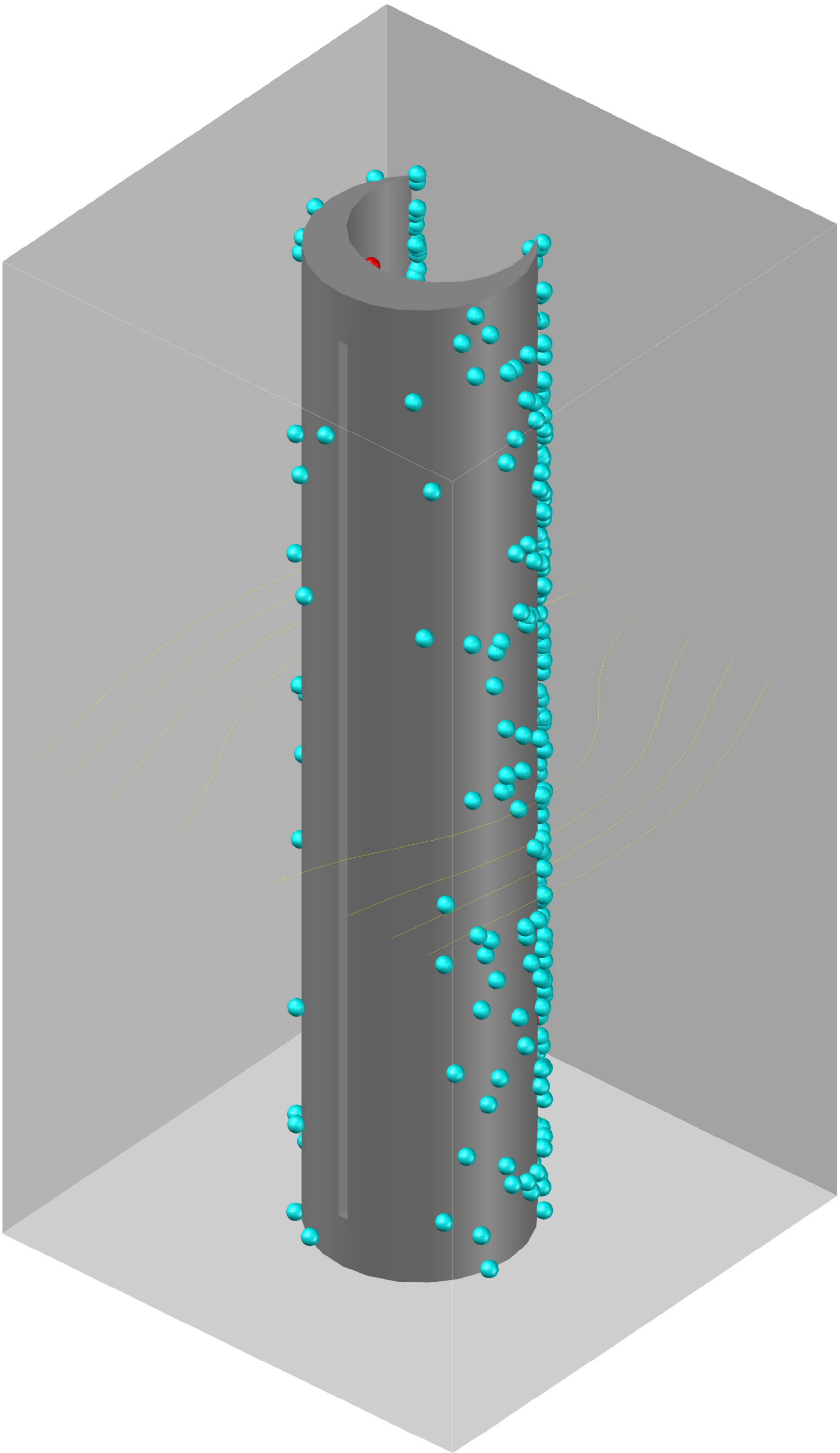

Fig 7-10. Visualizations of deposited particle distribution on the leeward of the slit-crescent-shaped fiber with $\delta = 0.05$ and $d_p = 0.5\mu m$.

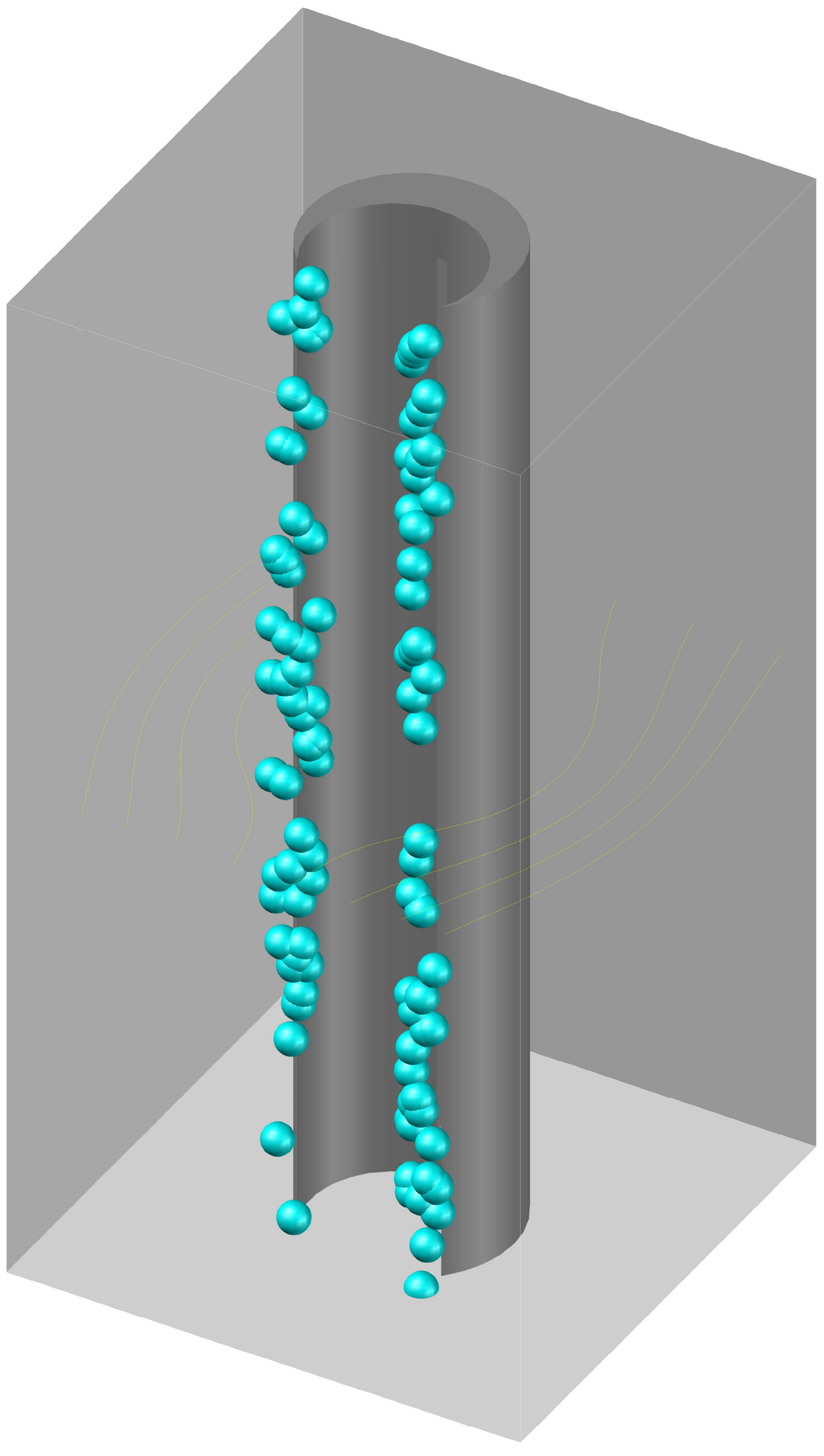

Fig 7-11. Visualizations of deposited particle distribution on the windward of the slit-crescent-shaped fiber with $\delta = 0.05$ and $d_p = 2.5\mu m$.

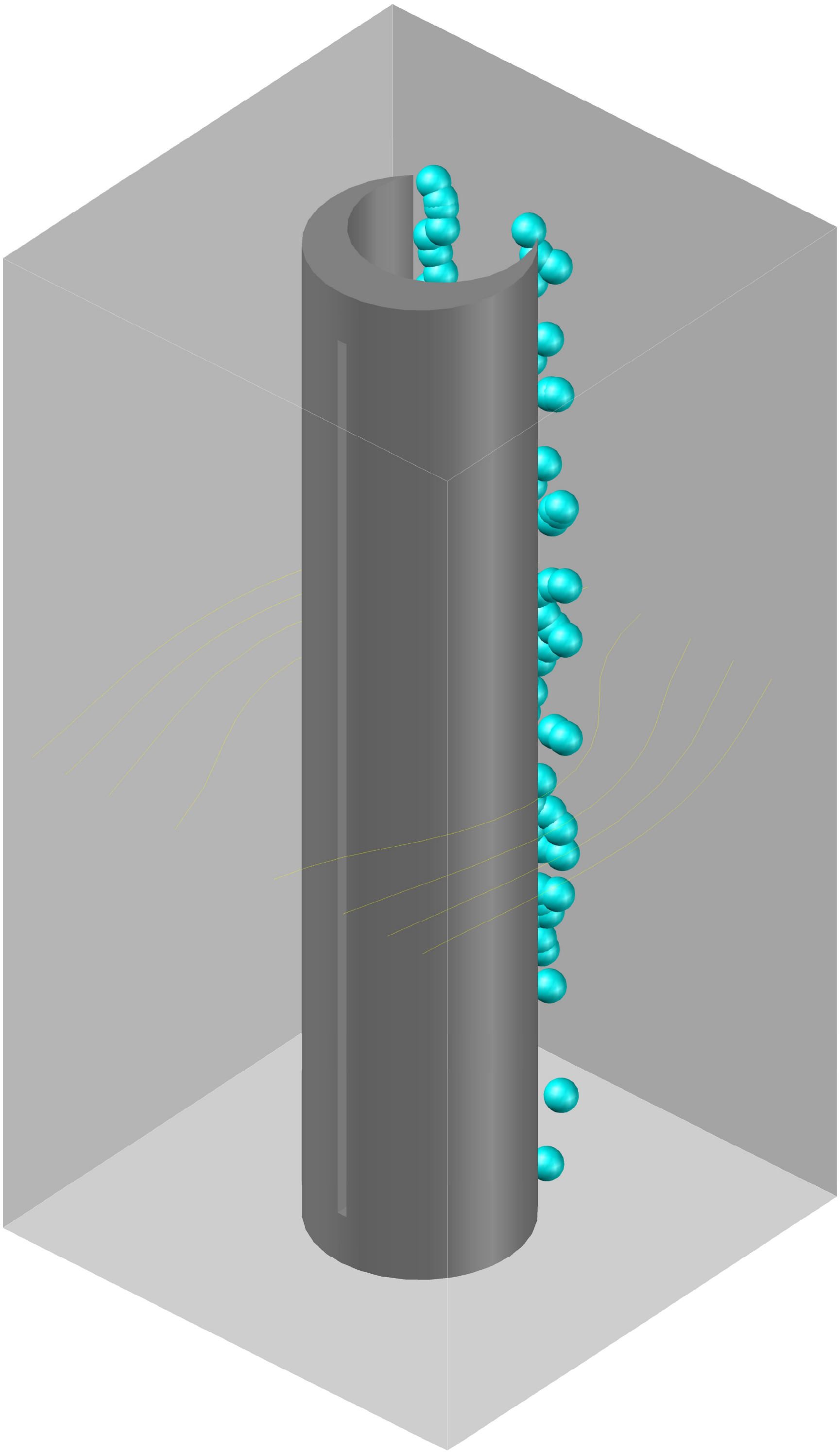

Fig 7-12. Visualizations of deposited particle distribution on the leeward of the slit-crescent-shaped fiber with $\delta = 0.05$ and $d_p = 2.5\mu m$.

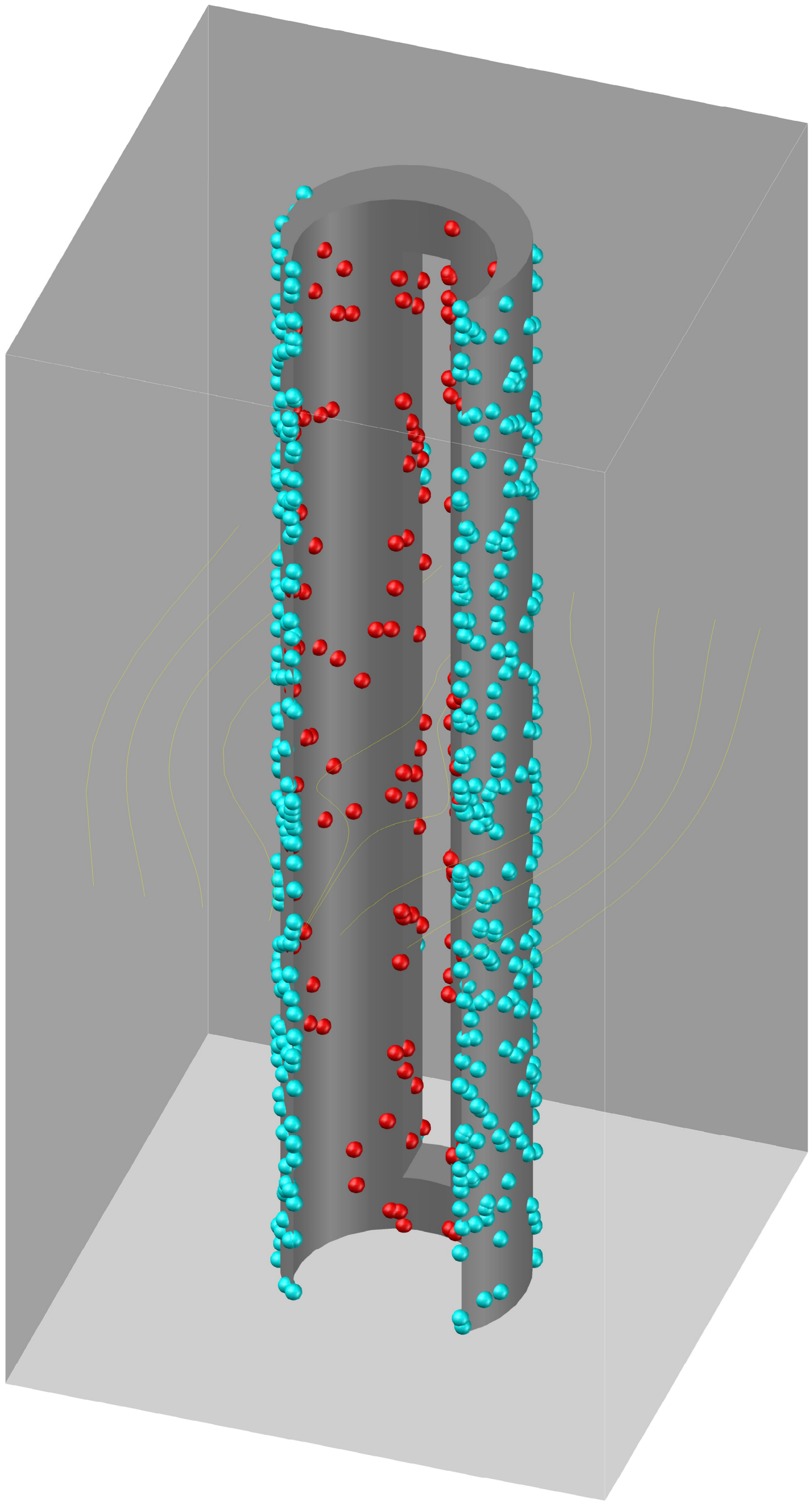

Fig 7-13. Visualizations of deposited particle distribution on the windward of the slit-crescent-shaped fiber with $\delta = 0.2$ and $d_p = 0.1\mu m$.

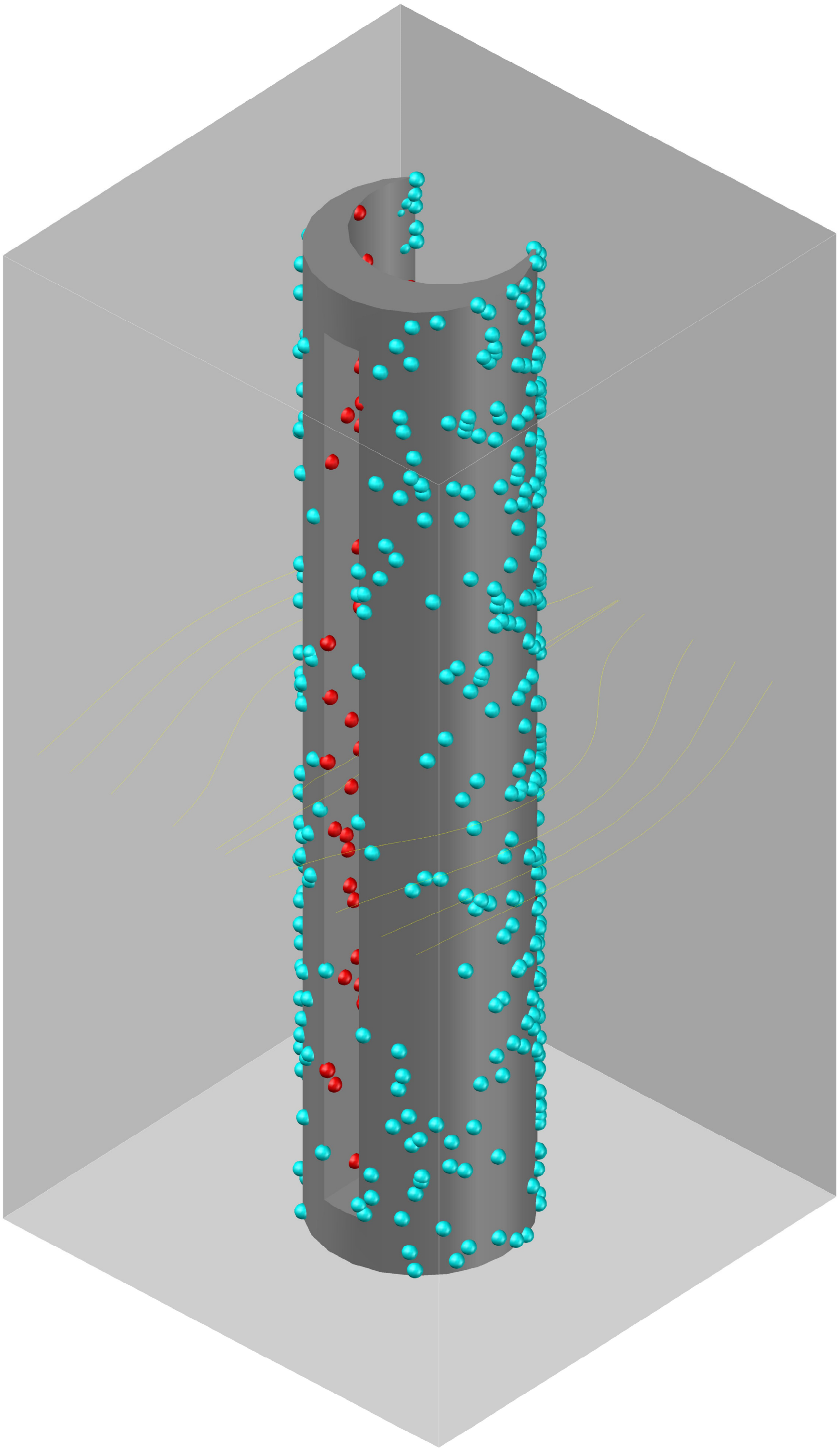

Fig 7-14. Visualizations of deposited particle distribution on the leeward of the slit-crescent-shaped fiber with $\delta = 0.2$ and $d_p = 0.1\mu m$.

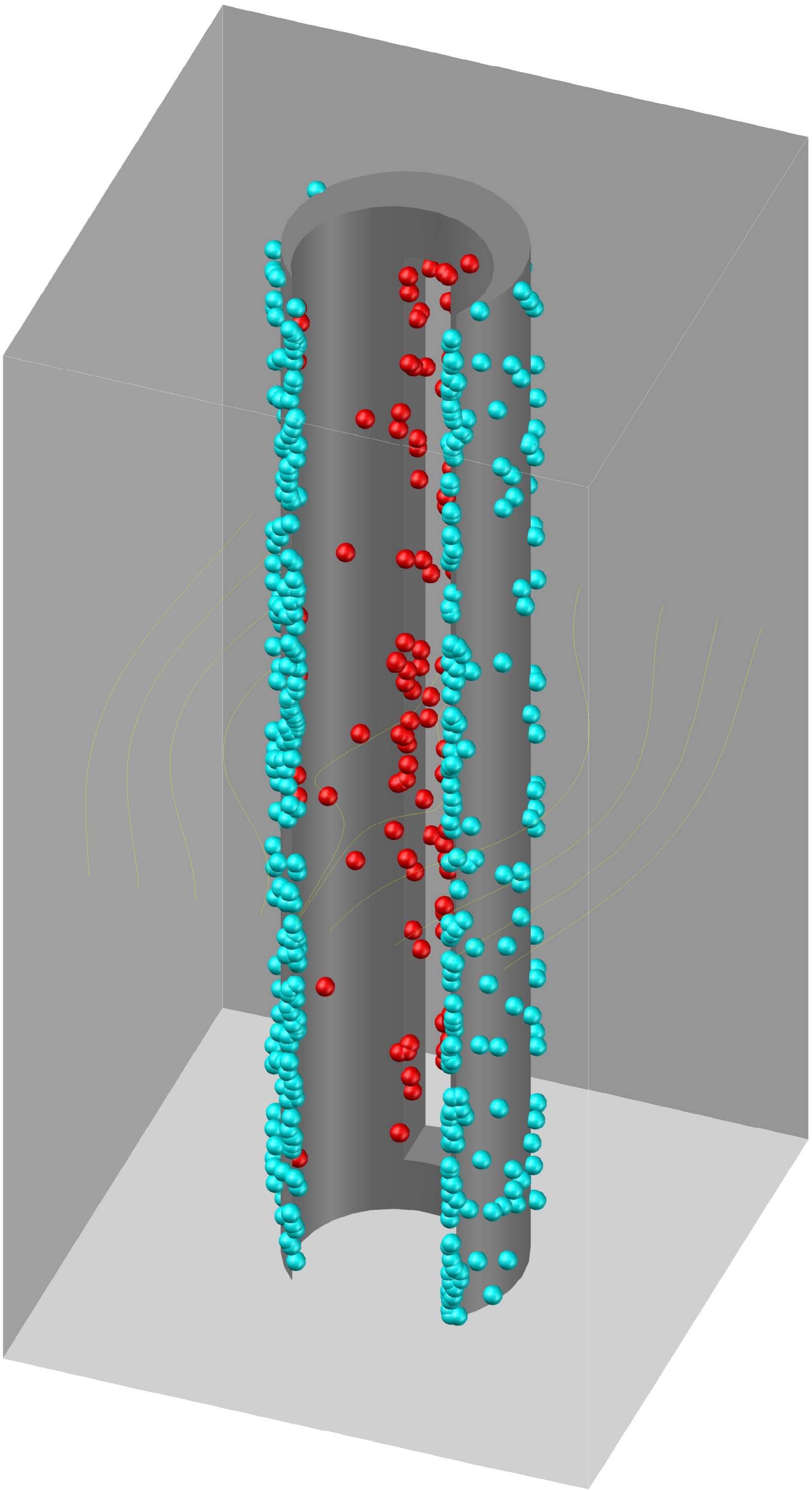

Fig 7-15. Visualizations of deposited particle distribution on the windward of the slit-crescent-shaped fiber with $\delta = 0.2$ and $d_p = 0.5\mu m$.

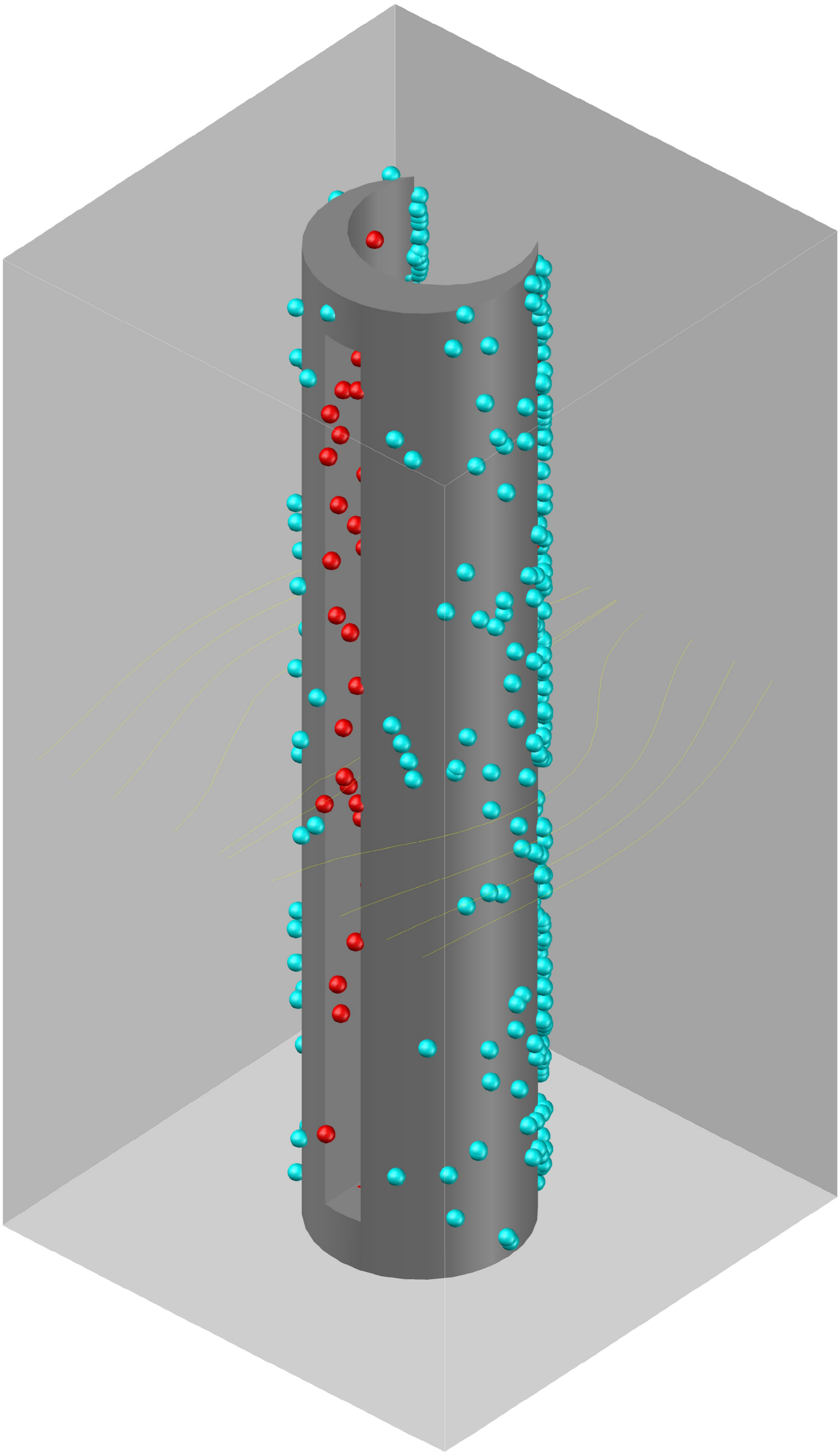

Fig 7-16. Visualizations of deposited particle distribution on the leeward of the slit-crescent-shaped fiber with $\delta = 0.2$ and $d_p = 0.5\mu m$.

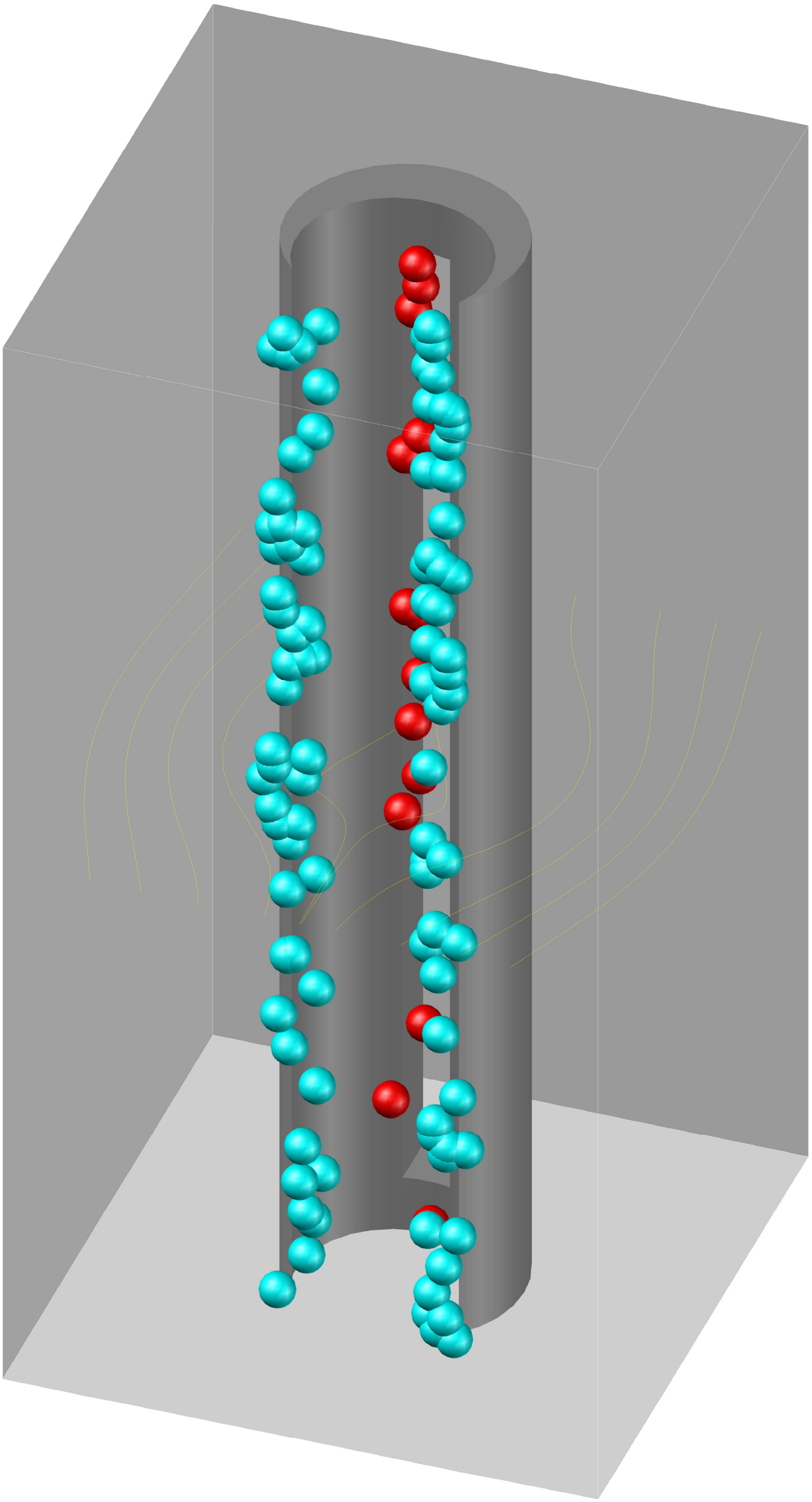

Fig 7-17. Visualizations of deposited particle distribution on the windward of the slit-crescent-shaped fiber with $\delta = 0.2$ and $d_p = 2.5\mu m$.

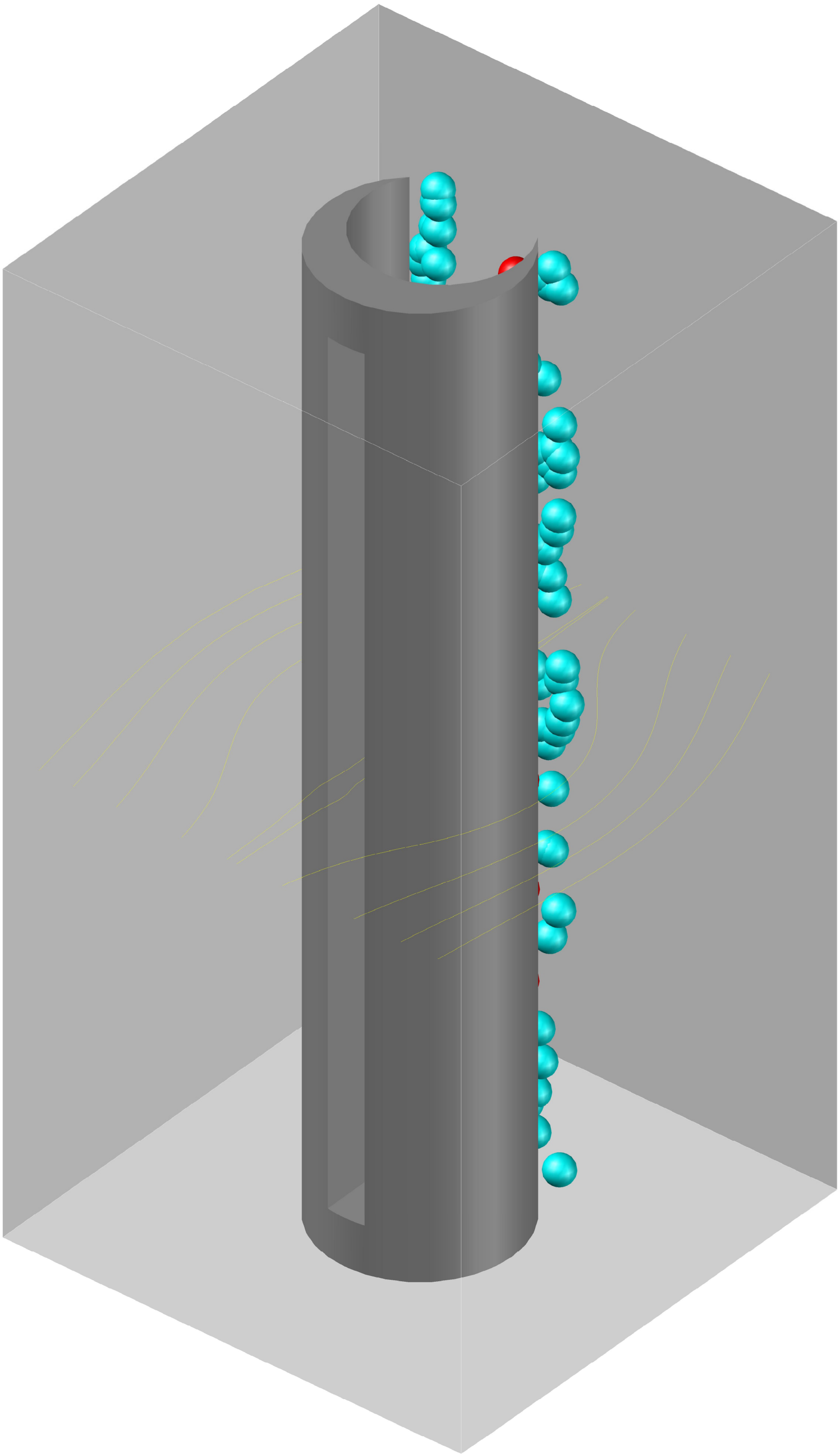

Fig 7-18. Visualizations of deposited particle distribution on the leeward of the slit-crescent-shaped fiber with $\delta = 0.2$ and $d_p = 2.5\mu m$.

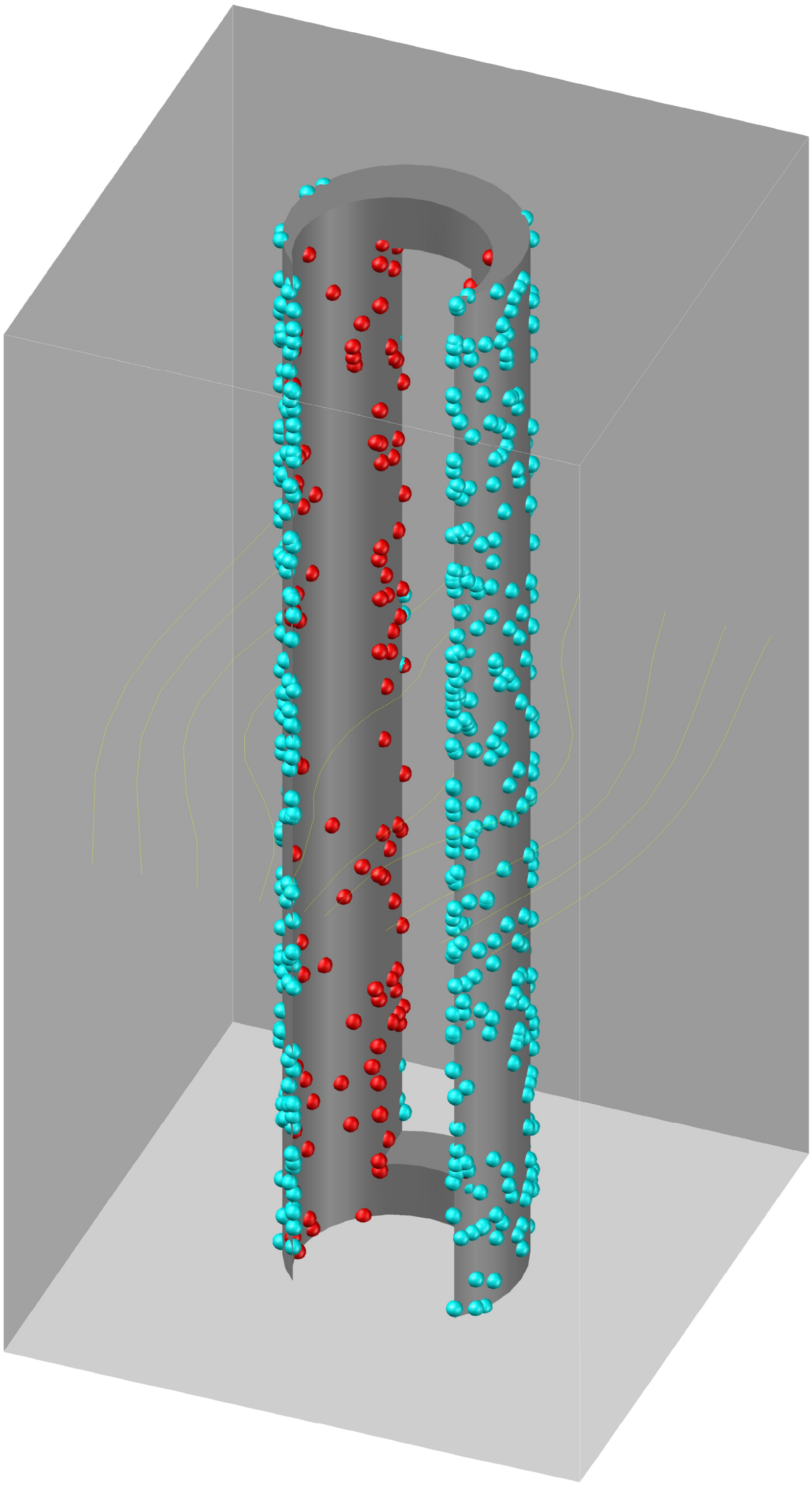

Fig 7-19. Visualizations of deposited particle distribution on the windward of the slit-crescent-shaped fiber with $\delta = 0.05$ and $d_p = 0.1\mu m$.

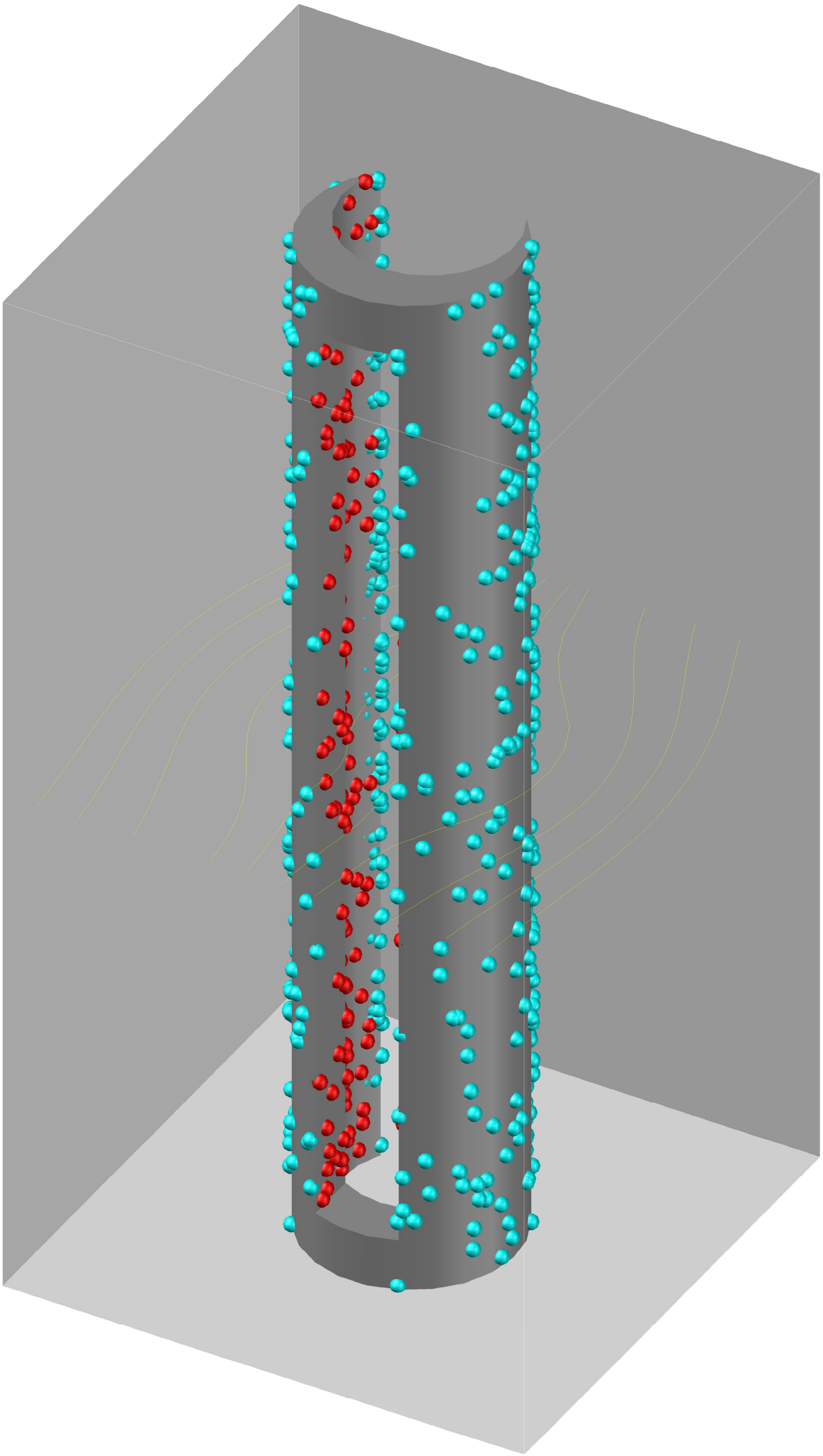

Fig 7-20. Visualizations of deposited particle distribution on the leeward of the slit-crescent-shaped fiber with $\delta = 0.05$ and $d_p = 0.1\mu m$.

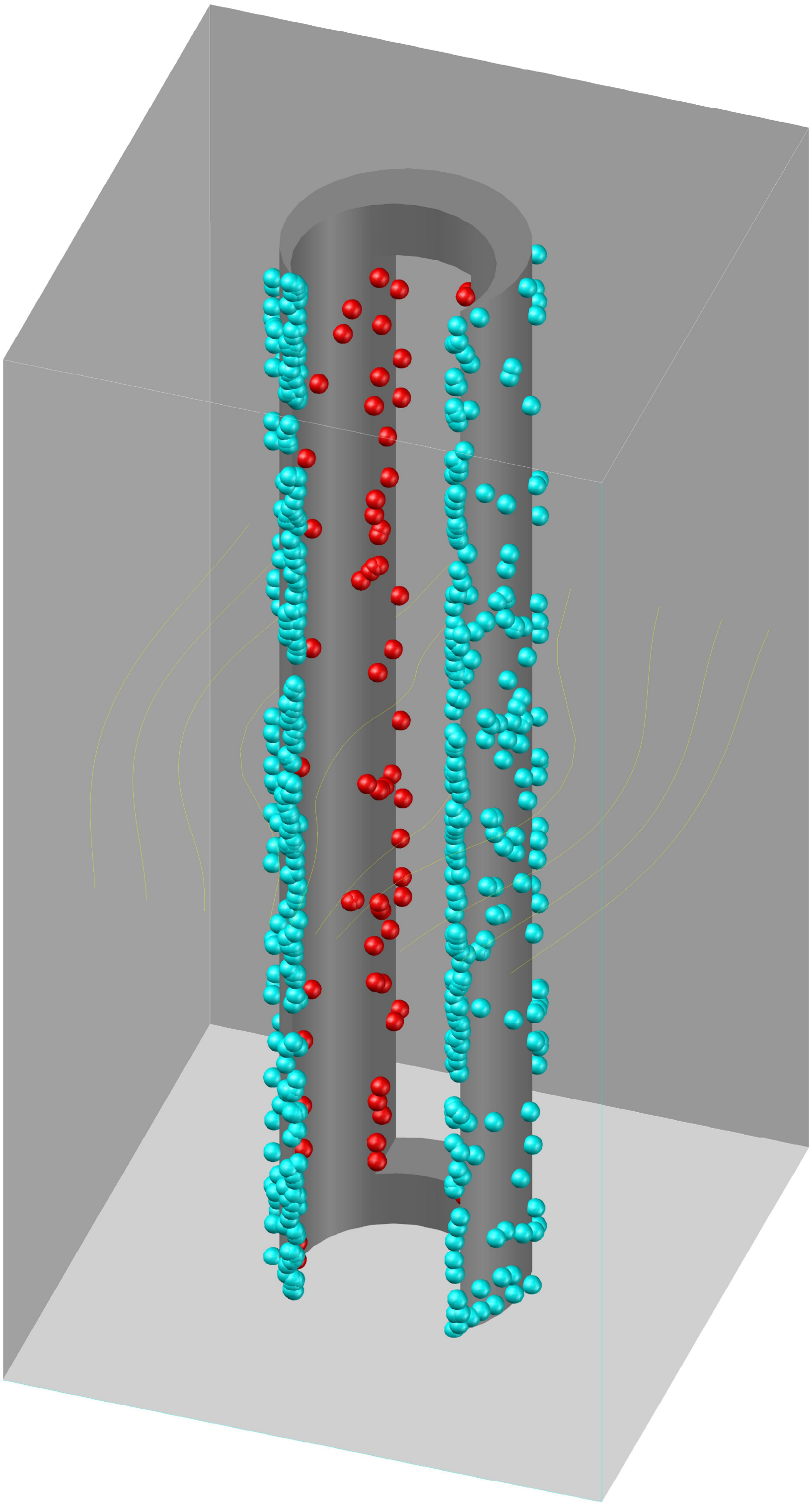

Fig 7-21. Visualizations of deposited particle distribution on the windward of the slit-crescent-shaped fiber with $\delta = 0.05$ and $d_p = 0.5\mu m$.

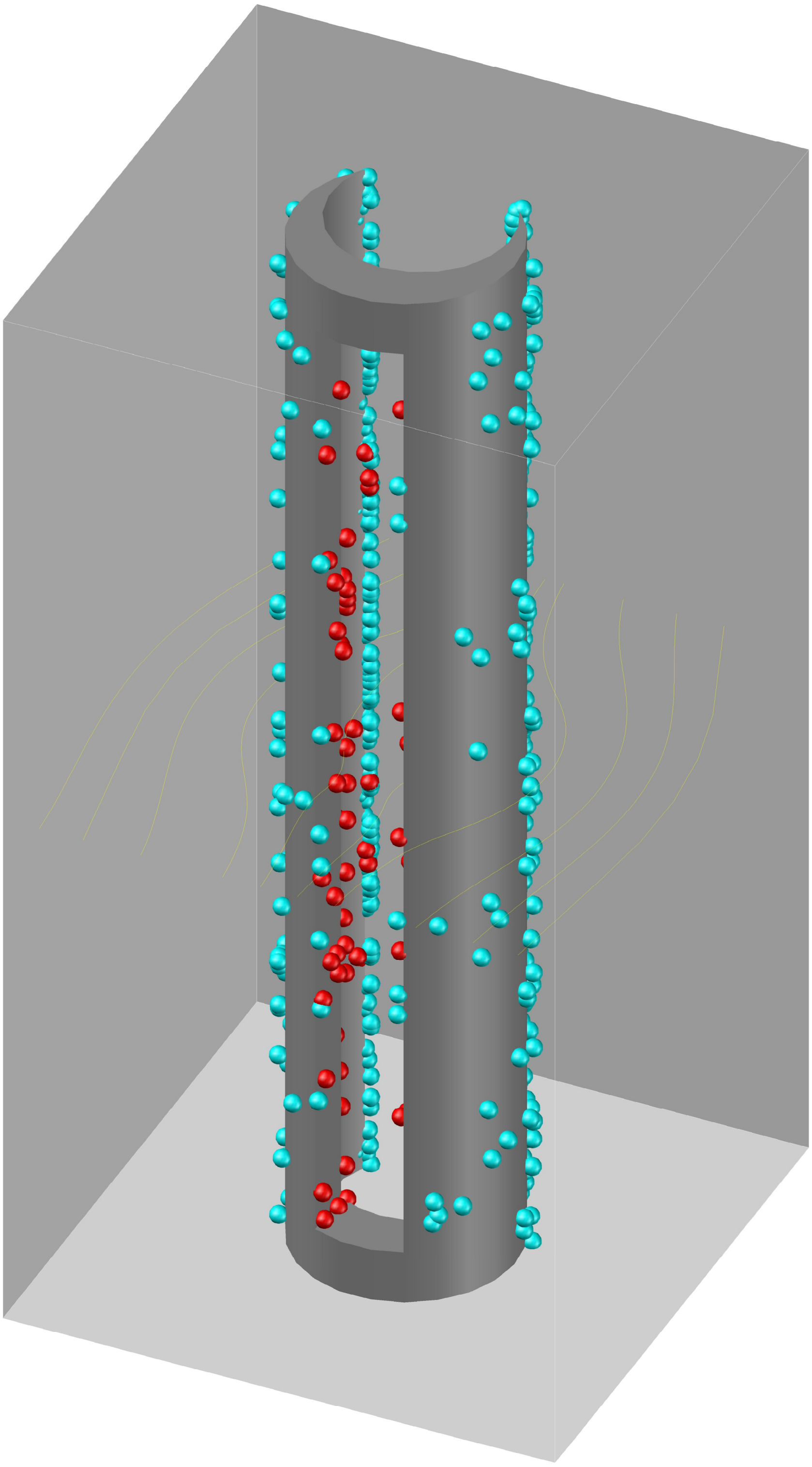

Fig 7-22. Visualizations of deposited particle distribution on the leeward of the slit-crescent-shaped fiber with $\delta = 0.05$ and $d_p = 0.5\mu m$.

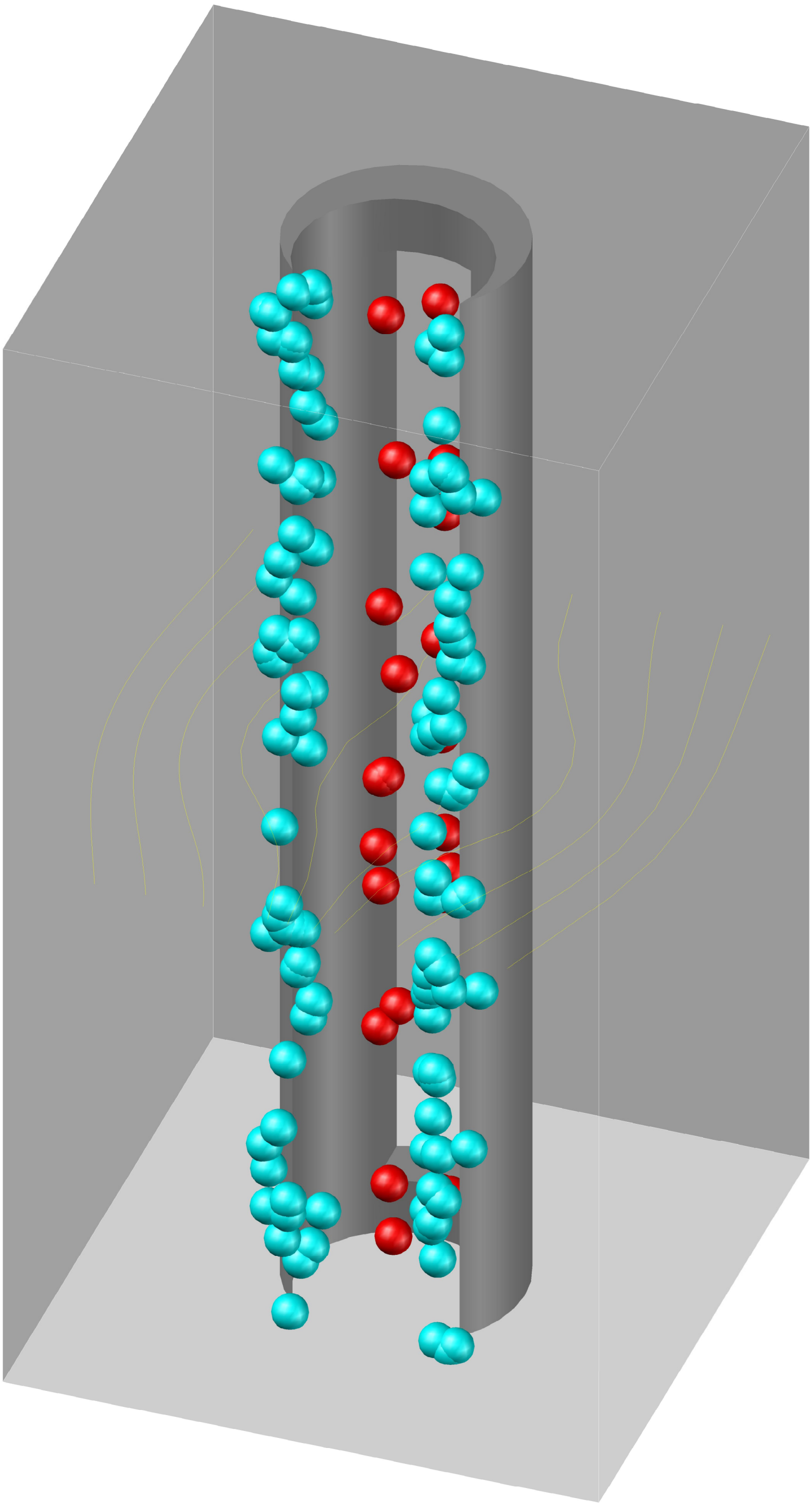

Fig 7-23. Visualizations of deposited particle distribution on the windward of the slit-crescent-shaped fiber with $\delta = 0.05$ and $d_p = 2.5\mu m$.

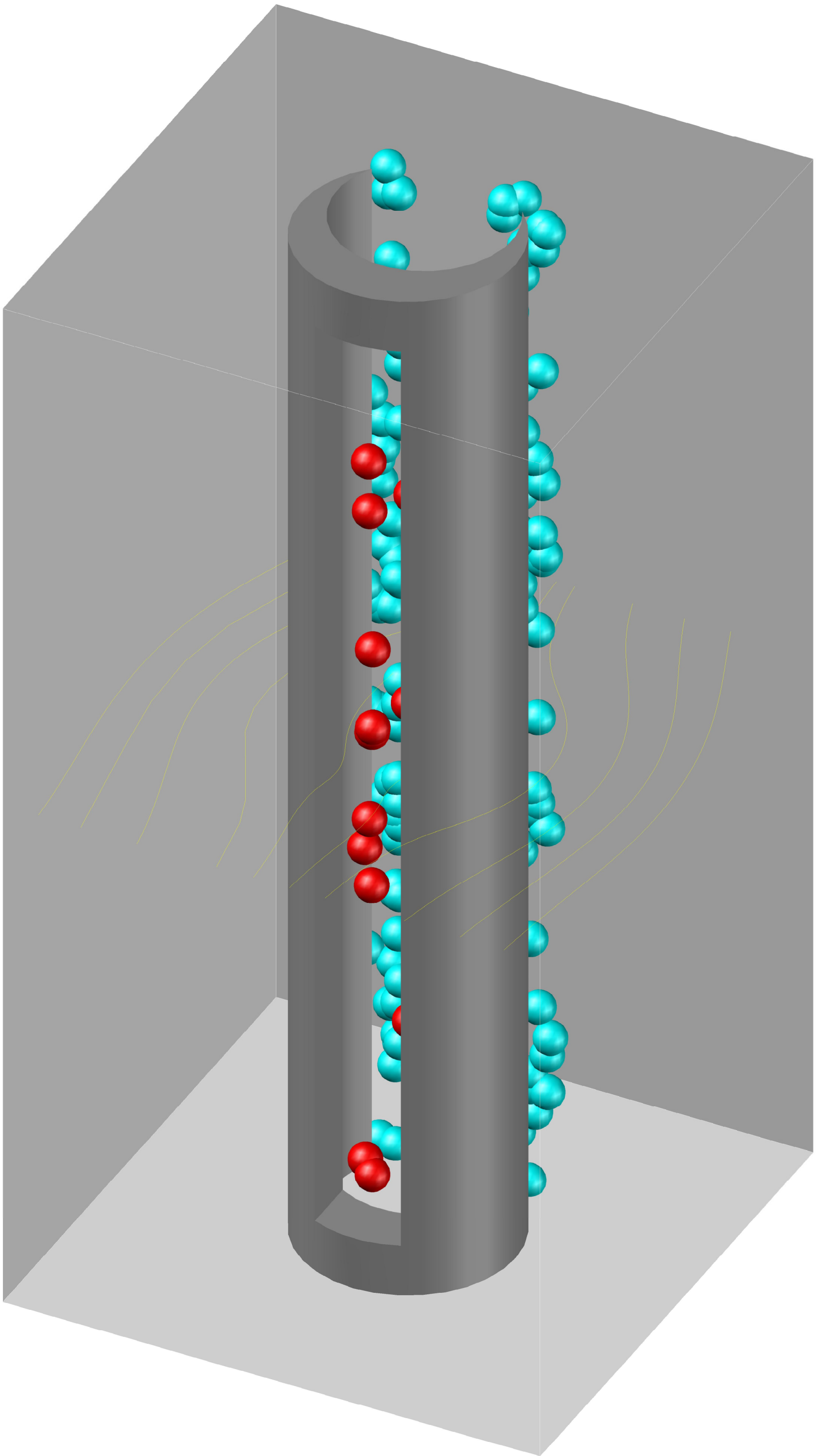

Fig 7-24. Visualizations of deposited particle distribution on the leeward of the slit-crescent-shaped fiber with $\delta = 0.05$ and $d_p = 2.5\mu m$.
